# An Amperometric Sensor with Anti-Fouling Properties for Indicating Xylazine Adulterant in Beverages

**DOI:** 10.3390/mi15111340

**Published:** 2024-10-31

**Authors:** Arielle Vinnikov, Charles W. Sheppard, Ann H. Wemple, Joyce E. Stern, Michael C. Leopold

**Affiliations:** Department of Chemistry, Gottwald Center for the Sciences, University of Richmond, Richmond, VA 23173, USA; arielle.vinnikov@richmond.edu (A.V.); charlie.sheppard@richmond.edu (C.W.S.); holly.wemple@richmond.edu (A.H.W.);

**Keywords:** xylazine, beverages, amperometry, sensors, carbon nanotubes, date rape, sexual assault

## Abstract

Amperometric electrochemical sensing schemes, which are easily fabricated and can directly relate measured current with analyte concentrations, remain a promising strategy for the development of the portable, in situ detection of commonly employed adulterants. Xylazine (XYL) is a non-narcotic compound designed for veterinary use as a sedative known as Rompun^®^. XYL is increasingly being abused as a recreational drug, as an opioid adulterant and, because of its chemical properties, has found unfortunate prominence as a date rape drug spiked into beverages. In this study, a systematic exploration and development of fouling-resistant, amperometric XYL sensors is presented. The sensing strategy features layer-by-layer (LBL) modification of glassy carbon electrodes (GCEs) with carbon nanotubes (CNTs) for sensitivity and the engagement of cyclodextrin host–guest chemistry in conjunction with polyurethane (PU) semi-permeable membranes for selectivity. The optimization of different materials and parameters during development created a greater fundamental understanding of the interfacial electrochemistry, allowing for a more informed subsequent design of effective sensors exhibiting XYL selectivity, effective sensitivity, rapid response times (<20 s), and low estimated limits of detection (~1 ppm). Most importantly, the demonstrated XYL sensors are versatile and robust, easily fabricated from common materials, and can effectively detect XYL at <10 ppm in both common alcoholic and non-alcoholic beverages, requiring only minimal volume (20 µL) of the spiked beverage for a standard addition analysis.

## 1. Introduction/Background

A derivative of the drug clonidine originally developed for the treatment of hypertension, xylazine (XYL) or N-(2,6-dimethylphenyl)-5,6-dihydro-4H-1,3-thiazin-2-amine, is a thiazine-class molecule that is currently not a scheduled drug or approved by the FDA for use in humans. Instead, this non-narcotic and very potent α2-adrenergic agonist is widely used in veterinary medicine as a sedative and analgesic [1,2,3,4]. Manufacturers supply veterinarians XYL under the drug names Chanazine (Chanelle Pharma, London, UK), Sedazine (Lloyd, Shanandoah, VA, USA), Rompun (Bayer, Leverkusen, DE, USA), Anased (Aspen Veterinary Resources, Liberty, MO, USA), and Proxylaz (Prodivet Pharmaceuticals, Raeren, Belgium ©) with a dosage determined by the animal’s drug sensitivity and as a function of their weight (i.e., per pound) [5]. These XYL drugs can be administered intravenously, subcutaneously, or intramuscularly [1,2,3,4]. XYL is useful in this capacity because of its relatively short half-life (3–4 h) after being quickly absorbed to yield analgesic effects for 30 min and sedative effects for ~2 h while it is being metabolized and cleared from the body [1,2].

As with many compounds of this nature, XYL has become an abused substance on a worldwide scale as a recreational drug. XYL emerged as an adulterant for opioids (e.g., heroin), narcotics (e.g., cocaine), and dissociative drugs (e.g., ketamine), where it allows for lowering illicit production costs without losing the euphoric effects of the adulterated compounds. In recent years, XYL is now also finding devastating prominence as an adulterant to fentanyl-based street drugs (e.g., “Tranq” or “Zombie”), responsible for the rapidly escalating overdose deaths across the globe [5,6,7]. The attraction of the mixture for drug addicts is that the XYL-doped fentanyl drugs extend the relatively short-lived “high” of fentanyl at the cost of significant increases in overdose deaths and other drug-related fatalities [8,9,10]. Additionally, intravenous users of XYL-adulterated fentanyl who inadvertently miss their veins during injection suffer severe skin ulcers and tissue necrosis [7,11].

An additional illicit use of XYL is in the adulteration of beverages for the purposes of sexual assault. The Drug Enforcement Agency recently emphasized the potential use of XYL in drug-related crimes such as date rape [12]. Being colorless, odorless, tasteless, and having a short half-life in the human body, XYL possesses the properties for an effective “date rape” drug that can be easily spiked into a beverage [4,13]. Depending on the level of exposure, XYL can cause drowsiness, muscle weakness, and sleep within minutes of ingestion and the symptoms can last for several hours. Once consumed in a beverage, XYL detection in bodily fluids becomes a timing challenge because of the efficiency with which it is cleared from the body [3,4]. One case study even implicated the use of xylazine in a sexual assault against a 4-year-old where xylazine was detected in the victim’s urine and blood samples [13]. First responders treating potential overdose victims can use antagonist compounds such as yohimbine, tolazoline, and atipamezole to minimize and/or reverse XYL effects but only if the consumption/presence of XYL can be quickly ascertained on scene [7]. XYL use in these types of crimes is often found to be an understudied application of XYL, and there are limited studies looking at sensor development for this particular application. Thus, from the potential victims of sexual assault to crime scene investigators/detectives, as well as for first responders to overdoses, a simple, affordable XYL detection system that can be easily used onsite by non-experts would be a significant development.

Forensic detection systems for targeted compounds take two primary forms known as either presumptive (i.e., screening) tests conducted in the field or confirmatory qualitative/quantitative analyses performed in a laboratory [14]. Like many illicit compounds, the latter involves the use of instrumentation, sample pre-treatment, and trained personnel—all of which extend the total analysis time, which can subsequently impact any investigation. In the lab, XYL can be definitively analyzed (i.e., with confirmational tests) in a mixture or within bodily fluids using a traditional instrumental analysis (e.g., chromatography, spectroscopy, or mass spectrometry) [1,3,4]. Presumptive field tests, on the other hand, while they can be inherently prone to some false positives, would be valuable for quick identification of compounds or classes of chemicals on-scene [15]. Examples of electrochemical and colorimetric XYL detection methods that lend themselves to field screening tests have been reported [1,2,3,4,16,17], with a limited subset of those reports specifically focused on XYL detection in beverages [3,4,16].

In 2019, Mendes and coworkers produced a foundational study examining the electrochemistry of XYL at glassy carbon electrodes (GCEs) using differential pulse voltammetry (DPV) for XYL quantification [1]. A critical development of this work was the recognition that while carbon-based interfaces like GCEs are sensitive to XYL oxidation, they are also easily fouled under potential control. That same year, El-Shal and Hendawy published one of the first known papers to employ carbon-based nanomaterials (NMs) at the electrode interface [2]—a strategy commonly used for signal enhancement in sensors [18,19,20]. In the El-Shal report, XYL was successfully detected using electrodes incorporating carbon nanotubes (CNTs) within an ionic liquid film [2]. Recognizing the need to quickly analyze XYL in beverages, Limbut and coworkers published two strong studies ultimately aimed at analyzing beverages using screen-printed electrodes modified with graphene nanoplatelets [4] and nanocoral-modified graphene paper-based electrodes [3]. These studies employed stripping voltammetry and DPV, respectively, to quantify XYL in certain beverages, though both methods resulted in calibration curves (CCs) with two different linear ranges (i.e., sensitivities) at low and high concentrations that were again attributed to electrode fouling [3,4]. More recently, laser-scribed graphene electrodes were presented by de Araujo that quantified XYL in synthetic urine as well as in a limited number of drinks, though the technique requires significant electrode fabrication and pretreatment [21,22]. In terms of non-electrochemical techniques, Marroquin-Garcia et al. published a clever colorimetric XYL detection system using molecularly imprinted polymers and dye displacement, though the limit of detection was determined to be 300 ppm of XYL [16].

The overall aim of our research program was the development of electrochemical sensors incorporating NMs for the detection of molecules relevant to a specific application, including presumptive screening methods. More specifically, in this report targeting XYL, the research goals included the development of a screening test that simplifies the materials involved, the electrode modification, the applied electrochemical technique, as well as offering the ability to use the sensor on beverage samples. To our knowledge, this work represents the only amperometry-based sensor targeting XYL in beverages. Prior work in the lab involved the development of amperometric uric acid sensors utilizing GCEs modified with layer-by-layer constructs of MWCNTs for sensitivity, cyclodextrins for engaging host–guest selectivity, and various semi-permeable membranes [23,24,25,26]. Given the simplicity of amperometric sensors and literature reports suggesting that host–guest chemistry exists between β-cyclodextrin (β-CD) and XYL [27,28], these materials were combined to create a XYL sensor targeting beverage analysis.

## 2. Experimental Details

### 2.1. Materials and Instrumentation

All solutions were prepared using ultra-purified water (18.2 MΩ∙cm). Polyurethanes (PUs) of hydrothane (HPU, AL25-80A) and Tecoflex (TPU, SC-80A) were obtained from AdvanSource Biomaterials (Wilmington, MA, USA) and Lubrizol (Cleveland, OH, USA), respectively. Nafion (Liquion solution LQ–1105 1100EW 5% wt.) was purchased from Ion Power, Inc. (New Castle, DE, USA) and diluted. Carboxylic-acid derivatized multi-walled carbon nanotubes (COOH-MWCNT) and β-cyclodextrin (β-CD) molecules were purchased from Nano Lab Inc. (Waltham, MA, USA) and Ambeed, Inc. (Arlington Heights, IL, USA). Electrochemical workstations (Models 1000B and 1030C potentiostats) and electrodes, including the materials glassy carbon electrodes (GCE), Ag/AgCl reference electrodes, and a platinum wire auxiliary electrode were obtained from CH Instruments (Bee Cave, TX, USA). Redox probe molecules of potassium ferricyanide and hexaamineruthenium (III) chloride were purchased from Oakwood Chemical (Estill, SC, USA) and Strem Chemical (Newburyport, MA, USA), respectively. Xylazine was purchased from Chem-Impex International (Wood Dale, IL, USA) and used as freshly prepared 50 mM standards in a 150 mM potassium phosphate buffered solution (pH = 7). Beverage solutions were purchased locally at supermarkets and ABC stores including Coca-Cola products, Barton London Extra Dry Gin (40%-vol EtOH), Lunazul Tequilia Blanco (40%-vol EtOH), Jamaican Rum (43%-vol EtOH), and Bowman’s Premium Vodka (40%-vol EtOH). Potential interferent chemicals used included aspartame (Millipore-Sigma/Supelco), phenylalanine (Supelco), Acesulfame (Supelco), caffeine, citric acid, sucrose, and glucose (Millipore-Sigma, St. Louis, MO, USA). Carbonated beverages like soda and diet soda were first decarbonated with agitation and neutralized to pH ~7 with dropwise additions of NaOH [1].

### 2.2. Sensor Fabrication and Performance

Procedural steps for sensor fabrication closely adhered to previously developed procedures [24]. In brief, GCEs were initially polished on cloth plates (Buehler, Lake Bluff, IL, USA) using subsequently smaller alumni powder (1.0, 0.3, and 0.05 µm) and water slurries, followed by extensive rinsing with ultra-pure water and drying with a stream of nitrogen gas. For modification, three solution mixtures were prepared: (1) COOH-MWCNTs (2 mg in 1 mL of ethanol), (2) β-CD (2 mg in 1 mL ethanol), and (3) HPU (100 mg in 5 mL solution of a 1:1 mixture of ethanol/THF). The MWCNTs and β-CD solutions were sonicated for 30 min while PU mixtures were vigorously stirred/sonicated overnight until dissolution was complete.

For the optimized sensor composition, the dry GCEs were modified via micropipette depositions, initially with COOH-MWCNT (7 µL), ensuring complete coverage of the carbon surface area, followed by β-CD (7 µL), and finally HPU solution (12 µL) with a drying time of 10 min between each added layer. The newly modified GCEs were soaked in 25 mL of the sample solution for ~15 min prior to applying a potential. With a continuously stirred sample, a potential ranging from +0.9 to +1.2 V was applied to both the modified and unmodified GCEs for 3600 s prior to the injection of standardized compounds (e.g., xylazine), typically at 200 s intervals until the total desired solution concentration was achieved. As with prior biosensor studies, sensor response times (tr-_95%_) were conservatively measured at a point when 95% of the amperometric response had transpired [29,30,31].

In assessing potential interferents, selectivity coefficients (K_j,sel_) were calculated as previously reported for individual species [29,31] with the following equation to allow for a conservative assessment of sensor selectivity:

(1) $$K_{j,sel}=\log\left(\frac{\frac{{\Delta I}_{j}}{C_{j}}}{\frac{{\Delta I}_{a}}{C_{a}}}\right)$$

where ∆I_j_ and ∆I_a_ are the measured currents for a specific interferent species (j) and analyte (a), respectively, each normalized to their corresponding concentrations (C_j_ and C_a_). Negative values of K_j,sel_ indicate that the interferent is inconsequential (i.e., no significant response compared to the analyte response) whereas species with near-zero or positive values are selected for by the sensor.

For standard addition analyses, spiked XYL solutions (25 mL) were amperometrically measured before being treated with at least three successive XYL standard injections (10 µL of 50 mM XYL standard) with an amperometric measurement recorded after each injection.

## 3. Results and Discussion

**Figure 1:**
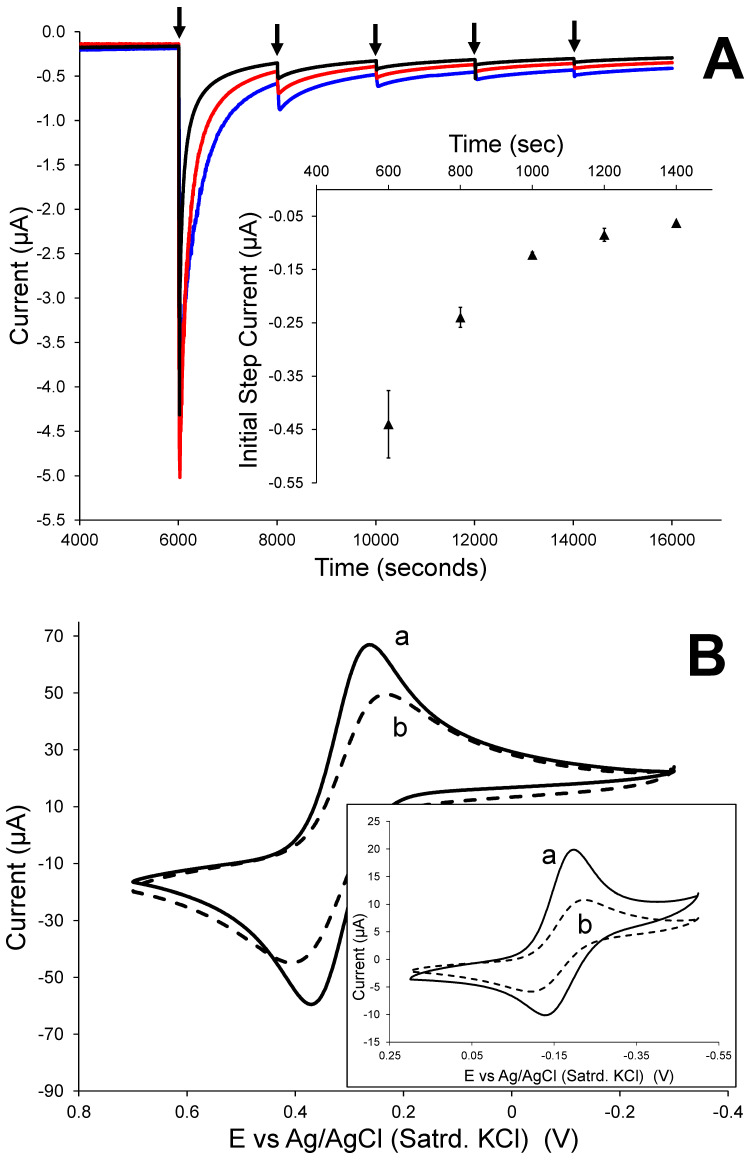
(**A**) Representative I-t curves during successive 50 µL injections of 50 mM XYL standard (**↓**) with the step current tracked over time (inset) with each 100 µM XYL increase and showing exponential decay of current signal. (**B**) Typical cyclic voltammetry of 5 mM potassium ferricyanide (1 M KCl) or 1 mM hexamine ruthenium (III) chloride (150 mM PBS) (inset) at (a) bare GCEs and (b) GCE after exposure to XYL under potential control (+1.1 V during 50 µL injections (5) of 50 mM XYL). Note: scan rate = 100 mV/s. Note: Different color scans represent replicate electrodes.

Most XYL electrochemical sensors must consider the known fouling issues that occur when electrodes, particularly those modified with carbon-based materials (e.g., graphene, CNTs, etc.), are subjected to positive, oxidizing potentials while XYL is introduced to the system [1,3,4]. Constant potential amperometry at +1.1 V, shown in Figure 1A as current-time (I-t) curves, represents an example of the fouling effect that occurs at unmodified GCEs with successive injections of XYL.

With each XYL injection into the stirred sample, the expected steady-state or stair-step response was not observed. Instead, diffusional-shaped spikes of current that exponentially decreased with each subsequent XYL injection were recorded (Figure 1A, inset). The GCE interface was probed using cyclic voltammetry (CV) on potassium ferricyanide (FeCN) and ruthenium hexamine (RuHex), both before and after this type of XYL exposure under oxidizing potential. The results (Figure 1B) illustrate the electrode fouling that causes subsequent electrode passivation and ultimately lowers sensitivity. This fouling phenomena of GCE with XYL has been previously reported in voltammetry-based studies that showed sensitivity was affected and, in some cases, required re-polishing electrodes prior to each exposure [1,4].

### 3.1. Sensor Fabrication and Optimization

**Scheme 1:**
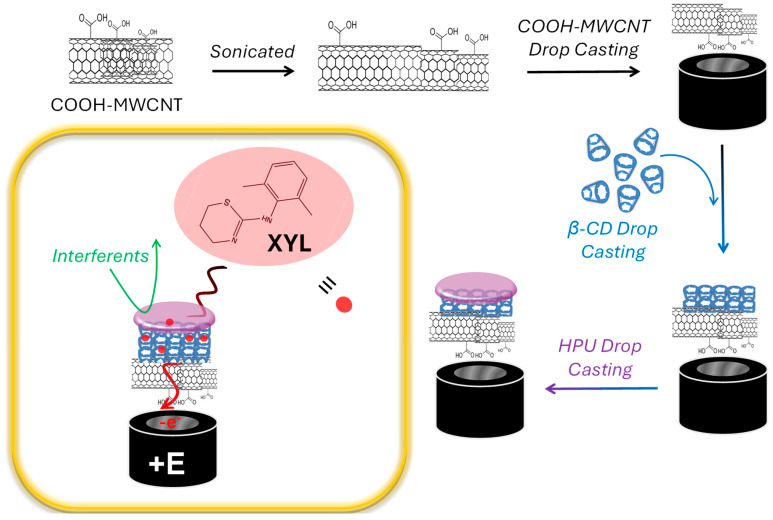
Step-wise schematic representation of the fabrication and operation (inset) of the modified GCE XYL sensor.

In fabricating the amperometric XYL sensors, GCEs were modified in a layer-by-layer manner as shown in Scheme 1 and detailed in the Experimental Section. The general approach involved the use of carbon-based NMs deposited on freshly polished GCEs before adding β-CD and capping the layers with the addition of a semi-permeable membrane of Nafion and/or a blended PU layer [24,32]. The NMs are added for enhancing sensitivity [24] while the other components are intended to improve selectivity for XYL [23,24,27,28]. While many of the materials and fabrication procedures were optimized as the first step of this study, other parameters were adapted from the lab’s prior work, including sonication times of certain NMs and the ratio of β-CD to CNTs, for example [24]. For this study, other parameters and materials were systematically examined for optimization using amperometry. Typically, these experiments involved five sequential injections of 50 mM of XYL standard (Figure 1A) while holding the electrodes at a constant potential (e.g., +1.1 V). The resulting I-t curve and the expected steady-state current responses were then evaluated for shape, magnitude, and resilience with increasing XYL concentrations. The XYL injection concentrations were purposefully high during the optimization experiments (i.e., a ~0.1–0.5 mM range or 200–1100 ppm) to observe clear trends occurring with specific layered materials.

**Figure 2:**
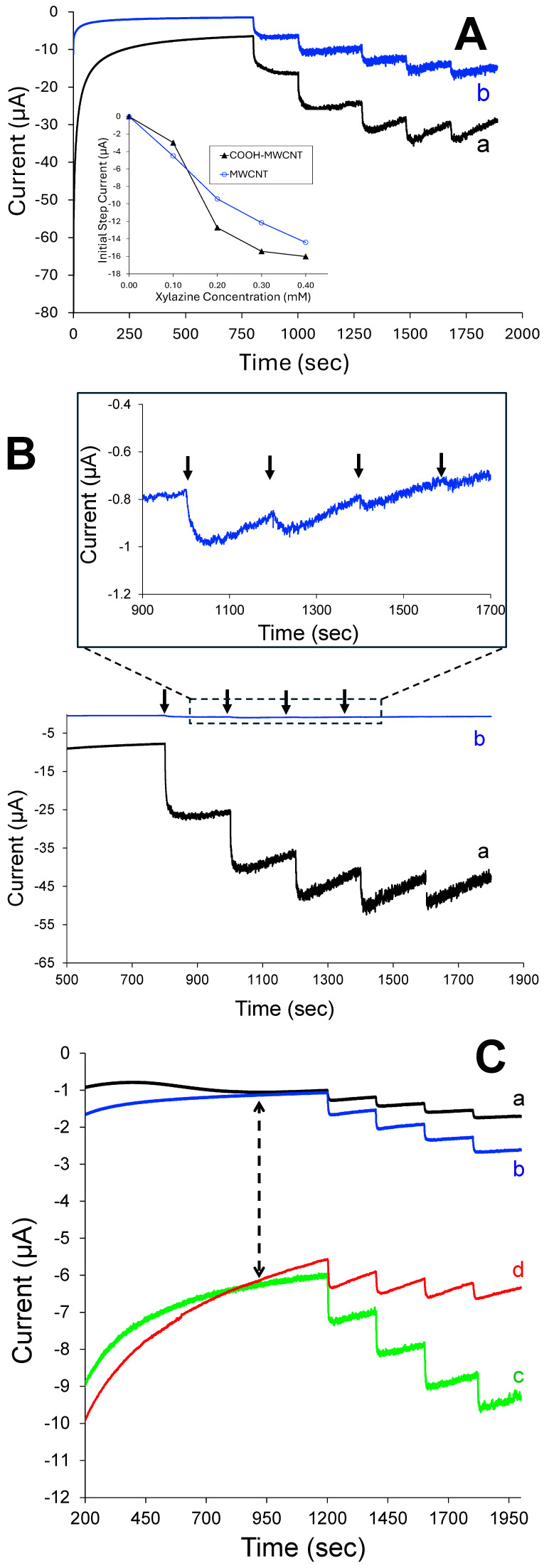
(**A**) Representative amperometric I-t curves during successive 50 µL XYL injections of a 50 mM XYL standard (100 µM increases) at GCE/MWCNTs/β-CD/Nafion featuring (a) COOH-MWCNTs vs. (b) MWCNTs and corresponding examples of step-current responses (inset). (**B**) I-t curves for (a) GCE/COOH-MWCNTs/β-CD/Nafion vs. (b) the same system without β-CD incorporated (expansion shows very small and diminishing stepping responses without β-CD). (**C**) I-t curves for fully-modified electrode (GCE/COOH-MWCNT/β-CD/PU (75:25) during (50 µL) injections of 50 mM XYL standard (100 µM increases) at different applied holding potentials: (a) +0.9 V, (b) +1.0 V, (c) +1.1 V, and (d) +1.2 V.

The materials and parameters optimized for the XYL sensor scheme included the following: (a) the type/treatment of carbon-based NMs and their functionalization, (b) the effect of incorporating β-CD, (c) the capping layer effects (Nafion vs. PU) on current response, including film thickness and composition, (d) the effect of sonication and layering, and (e) the applied potential. Several carbon-based NMs were considered for the sensor including single-walled carbon nanotubes (SWCNTs as received or sonicated), multi-walled carbon nanotubes (MWCNTs as received or sonicated), and with or without carboxylic acid functional groups and carbon nano-horns (as grown versus oxidized). To determine the most functional NMs, they were each deposited on GCEs followed by the addition of β-CD and a capping layer of Nafion (0.5%) [24]. I-t curves for the different NMs and the corresponding preliminary calibration curves (CCs), provided in Supporting Information (Figure S1), showed that MWCNTs functionalized with carboxylic acid (COOH-MWCNTs) produced the highest current response and exhibited partial resistance to fouling during XYL injections. An example of the evaluation of different types of MWCNT, pristine (p-MWCNT) versus COOH-MWCNTs, is shown in Figure 2A. Comparing I-t curves to the bare GCEs (Figure 1A), they showed enhancement in current and a more defined stair-step response with MWCNTs incorporated into the sensing scheme. While not specifically identified, we believe that the COOH-MWCNTs allowed for greater surface area after sonicating the carbon nanomaterials [33,34], coupled with carboxylic acid functionalities allowing for better integration with the aqueous media. Based on these results and prior work in the lab, e.g., ref. [24], COOH-MWCNTs were selected as the optimal XYL sensor.

A critical part of an optimized XYL sensor is evaluating the components providing selectivity, including the outer semi-permeable membranes (i.e., Nafion and PU) as well as the host–guest chemistry of β-CD [27,28]. In the latter regard, GCEs modified with COOH-MWCNTs and capped with Nafion showed considerable enhancements with the inclusion of β-CD (Figure 2B). When Nafion and PU were compared as outer layers, keeping the GCEs modified with COOH-MWCNTs and β-CD, PU generally produced more substantial and well-formed stepping for the five injections of XYL (Supporting Information, Figure S2). As such, numerous experiments were devoted to optimizing the blended PU layer in terms of the composition as it relates to XYL permeability and PU film thickness (i.e., deposition volume). As described in the Experimental Section, the blended PU layer is a mixture of hydrophilic (HPU) or hydrophobic (TPU) polymers [29,30,31,32]. To assess XYL permeability, bare GCEs (without COOH-MWCNTs or β-CD) were coated with different blends of PU and subjected to XYL injections. The results, provided in Supporting Information in Figure S3, show that XYL permeability is more considerable when a greater hydrophilic PU component (e.g., either 100:0 or 75:25 for HPU/TPU ratios) is utilized. In similar fashion, bare GCEs coated with different volumes of 75:25 HPU/TPU showed the most defined stair-step current response with 12 microliter depositions (Supporting Information, Figure S4). Different PU blends capping the fully modified GCEs with COOH-MWCNTs and β-CD also showed well-defined steps during XYL injections with high HPU/TPU ratios (Supporting Information, Figure S5).

A well-established pretreatment of MWCNTs is sonication prior to use as it increases both their dispersity and surface area during the modification of an electrode [24,33,34]. One of the parameters optimized in this study was the sonication and deposition of both COOH-MWCNTs and β-CD in a layer-by-layer fashion or as a single combined (i.e., mixture) deposition. The I-t curves and corresponding preliminary CCs of these films capped with a 75:25 PU blend layer showed that the current response was improved when the materials were deposited in two distinct layering steps (Supporting Information, Figure S6).

One of the more interesting parameters explored for the XYL sensor was its dependence on the applied potential. Prior to fouling, CV scans of a bare GCE in XYL showed oxidation occurring at +0.95 V (Supporting Information, Figure S7). As such, most of the optimization experiments were run at +1.1 V to ensure XYL oxidation. However, as seen in the I-t curves shown in Figure 2C, when the electrodes modified with optimal materials were run at different applied potentials (i.e., +0.9, +1.0, +1.1, or +1.2 V), the high positive potentials resulted in significantly larger charging currents which required longer wait times to stabilize to correct for the sloping stepping current responses. The corresponding current tracking during amperometry at different applied potentials is provided in Supporting Information, Figure S8. Notably, the potential dependence of the XYL sensor became a critical property to understand during application testing of the sensing scheme (see Section 3.3).

### 3.2. XYL Sensor Performance

**Figure 3:**
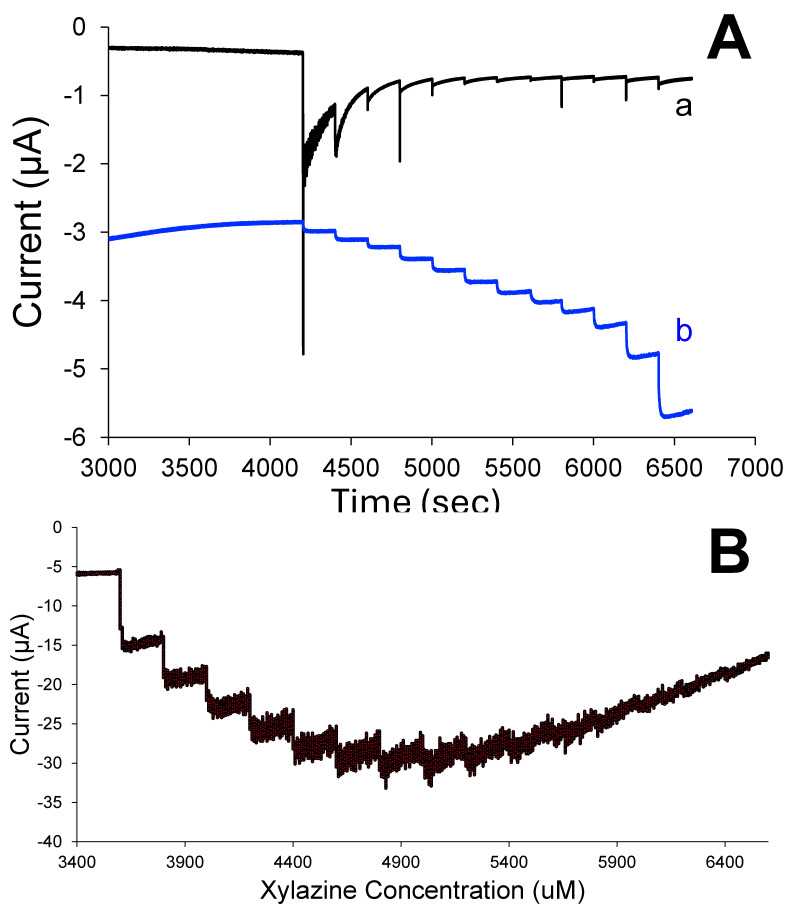
(**A**) Representative amperometric I-t curves during successive varying volume (0.5 to 100 µL) XYL injections of a 50 mM XYL standard for a concentration range of 1 to 605 µM XYL at (a) a GCE/MWCNTs/β-CD/HPU modified electrode versus (b) an unmodified GCE electrode. (**B**) I-t curve during similar injections at a modified electrode system without the HPU capping layer.

Optimized versions of the XYL sensors were used to detect lower concentrations of XYL that are more relevant to eventual applications. A striking comparison of the I-t curves in Figure 3A captures the fouling-resistant nature of the optimized modified electrodes. In these results, the optimized system, GCEs modified with COOH-MWCNTs, and β-CD with a PU capping layer (100:0) were compared to the bare electrode response during an injection of XYL. As expected, the bare GCE did not exhibit the expected stair-step response, and the current signal quickly diminished with increasing exposures to XYL. In stark contrast, the modified electrode displayed well-defined and consistent stepping beginning at 35 µM (~8 ppm), which was maintained until 600 µM (~130 ppm). The steady-state responses at increasing concentrations indicate that the sensor remained sensitive toward XYL and resisted passivation from fouling. As seen in Figure 3B, the PU layer appeared to be a critical component of the fouling resistance property by protecting the underlying COOH-MWCNT and β-CD components. In this result, the same electrode modification as in Figure 3A, but without the PU capping layer, initially stepped but was inconsistent, exhibiting an upward sloping step response, as well as an inevitable diminishment of signal/current.

**Figure 4:**
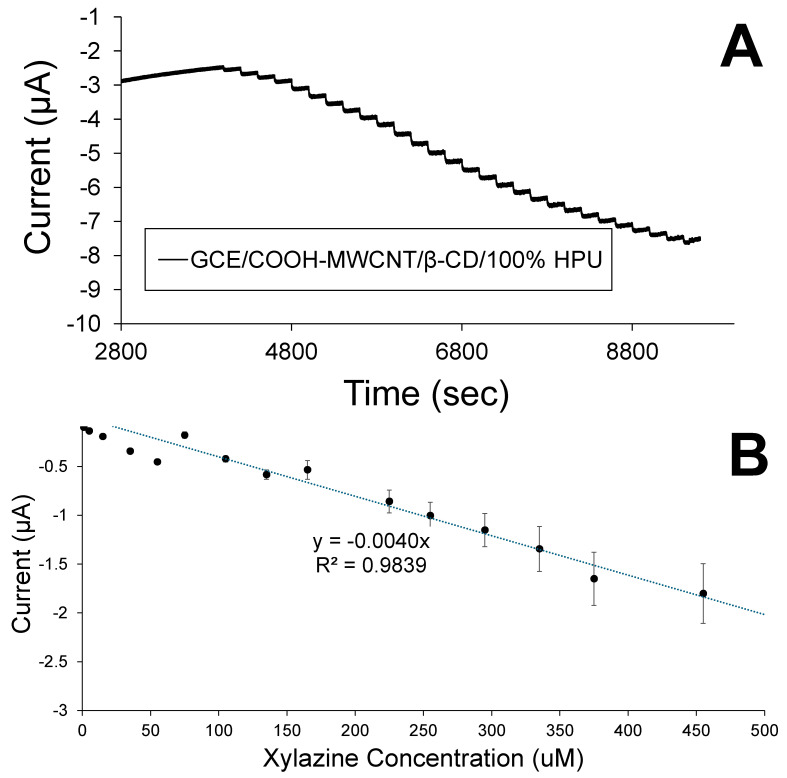
(**A**) Example of an amperometric I-t curve during successive varying volume (0.5 to 20 µL) XYL injections of a 50 mM XYL standard for a concentration range of 1 to 1015 µM showing stair-step responses at a modified electrode (GCE/COOH-MWCNT/β-CD/HPU), and (**B**) a calibration curve (1 to 450 µM) generated from amperometric responses from multiple (n = 16) modified electrodes. Note: a calibration curve spanning the larger concentration range is provided in Supporting Information; in both cases, the standard error estimate may be smaller than the average marker.

Figure 4A shows a representative I-t curve example of the consistency of the stair-step current response to XYL exposure by GCEs modified with the optimized system. Stepping responses could be recorded to 1015 µM (~225 ppm), a remarkable dynamic range compared to the bare GCE performance. The response time (t_r-95%_) of the sensor was fast upon XYL injection, conservatively estimated at an average of only 11.3 (±2.8) s, one of the major advantages of amperometric sensors. Figure 4B represents the corresponding CC analysis from the collected I-t curves using the optimized sensors and indicates a linear range between 0 and 450 µM (~100 ppm), an average sensitivity of ~5 nA/µM XYL, and limit of detection (LOD) conservatively projected to be <1 ppm based on the IUPAC definition (3∙σ_blank_/slope) [35]. The primary finding with the CC collected in the PBS was that there was a linear regression function between the signal (i.e., stepping current) and the XYL concentration, allowing for quantitative analysis techniques to be applied using these sensors.

### 3.3. XYL Detection in Real Beverages

**Figure 5:**
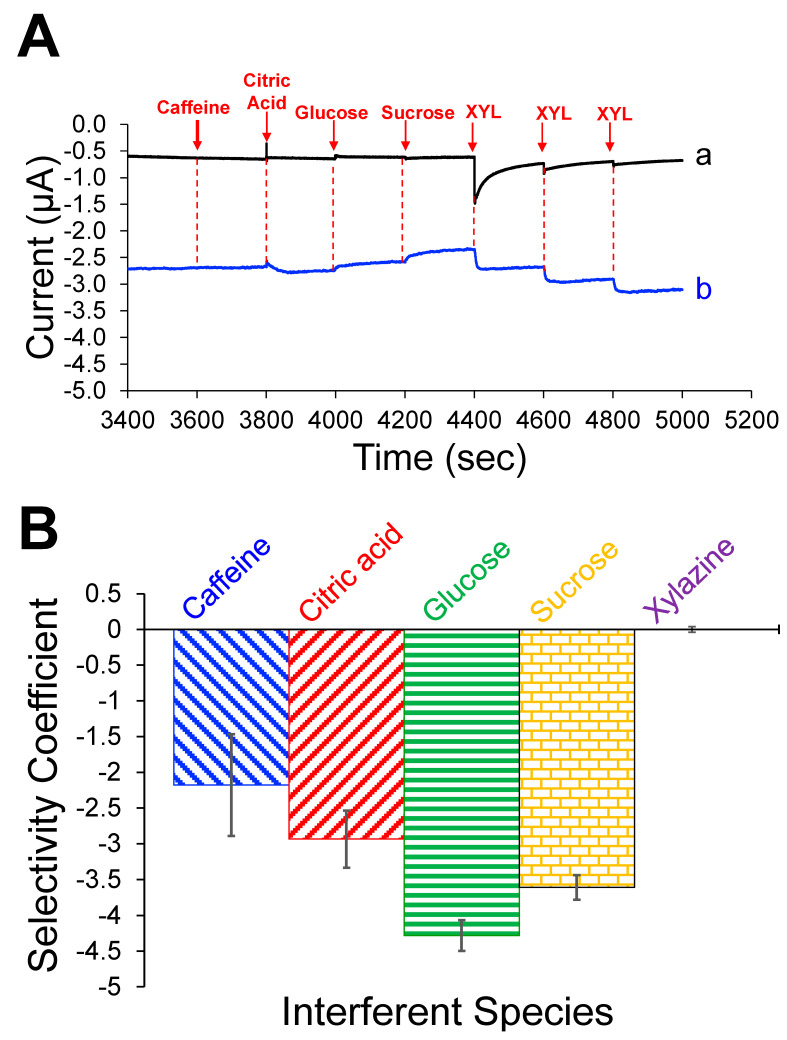
(**A**) Amperometric I-t curves during XYL injections (50 µL injections of 50 mM XYL standard) and potential interferent species in soda including injections resulting in 0.49 mM caffeine, 8.98 mM citric acid, 257 mM glucose, and 139 mM sucrose at (a) bare and (b) modified GCEs. (**B**) Corresponding graph of calculated selectivity coefficients for the potential interferent species at the modified GCE.

As with most amperometry sensor developments, an important step prior to applying the scheme to real samples is to identify and test the systems against specific interferent species and test sensor performance in a simplified version of the targeted samples. This preliminary step proved to be insightful about how the sensors can be applied. For this study, initial targets were soda and diet soda beverages due to their popularity and prominence at parties or as a component of mixed drinks. As part of a systematic approach, and as performed in prior reports [24,36], I-t curves were collected during injections of potential interferent species (j) known to be at significant concentrations in these beverages followed by an XYL standard at optimized sensors in order to calculate selectivity coefficients (K_j,sel_) (see ‘Section 2’). The potential interferents for soda included caffeine, citric acid, glucose, and sucrose while diet soda may contain other species including aspartame, Acesulfane K, and phenylalanine in addition to caffeine and citric acid. Figure 5A shows representative examples of I-t curves collected during the injection of the soda interferents as well as XYL at both a bare GCE and the developed sensor (i.e., fully modified electrode). Notably, the interferent responses were minimal at both interfaces. The XYL injection response at the two interfaces continued to contract each other with a clean stair-step current response at the modified electrode and a diffusional and successively fouled response at the bare GCE. Figure 5B is a graphical representation of the selectivity coefficient values collected from the interferents and XYL at the modified electrode. Species with negative selectivity coefficients were effectively discriminated against while those with positive or near-zero values, like XYL, were selected for by the modified electrode. Analogous results for diet soda are provided in Supporting Information (Figure S9). XYL selectivity was again demonstrated in the presence of common diet soda interferent species such as aspartame, Acesulfane K, and phenylalanine.

For the quantitative analysis of XYL, solutions of simulated soda and diet soda containing all the expected interferents were spiked with XYL and analyzed with the newly modified electrodes against a CC created from XYL standards in PBS (Figure 4) using a holding potential of +1.1 V during amperometric measurements. For both the simulated soda and diet soda solutions, however, the XYL spike was overestimated against the CC collected in PBS and resulted in high and widely varying percent recoveries of 167 (±50)% and 234 (±51)%, respectively. Suspecting that additive effects were causing the inaccuracy, standard CCs were generated by calibrating the electrodes in the simulated beverage solutions themselves (vs. PBS). The representative I-t curves collected at a hold potential of +1.1 V and the corresponding CC for the simulated soda and diet soda solutions are provided in Supporting Information (Figures S10 and S11) and continue to show a linear relationship between the signal and XYL concentration. That said, some percent recovery values suffered poor precision and accuracy (results not shown) and prompted the reevaluation of applying the sensors to actual beverage testing.

For actual beverage samples of soda and diet soda, a number of aspects of the application required strategic adjustment. Most notably, two aspects were further explored in how the sensors were executed in actual beverage samples: (1) the dependence of applied potential and (2) the presence of matrix effects. The magnitude of the applied potential, previously identified in Figure 2C exhibited two consequential effects of applying higher oxidizing potentials (e.g., +1.1 V) including a significant increase in charging current and more positively sloping stair-step current responses (i.e., a poor stair-step response). While the modified electrodes were fouling-resistant, they were not anti-fouling. As such, it was hypothesized that the exposure of the sensing interface to XYL oxidation at higher overpotentials in both magnitude, duration (time), and XYL concentration are likely to partially foul the electrodes to different extents and lead to biased measurements and inaccurate percent recoveries.

The second factor, the matrix effects of beverages, also needed to be addressed. The calibration of the electrodes in the actual sample matrix (soda and diet soda), referred to herein as conditioned electrodes, again produced a stair-step current response and linear CCs (Supporting Information, Figures S12 and S13). This approach also led to reasonable average percent recoveries of 80–90% with new sensors with the application of the standard curve, but electrode-to-electrode variability remained high. From the collective results using new electrodes to measure XYL in the different soda and diet soda samples (simulated and real), it was highly suspected that matrix effects were contributing to the varying percent recoveries. The application of sensors to real-world samples is often complicated by their matrices and the most traditional approach is to both condition the electrodes and employ standard addition (SA) methodology [1,37].

**Figure 6:**
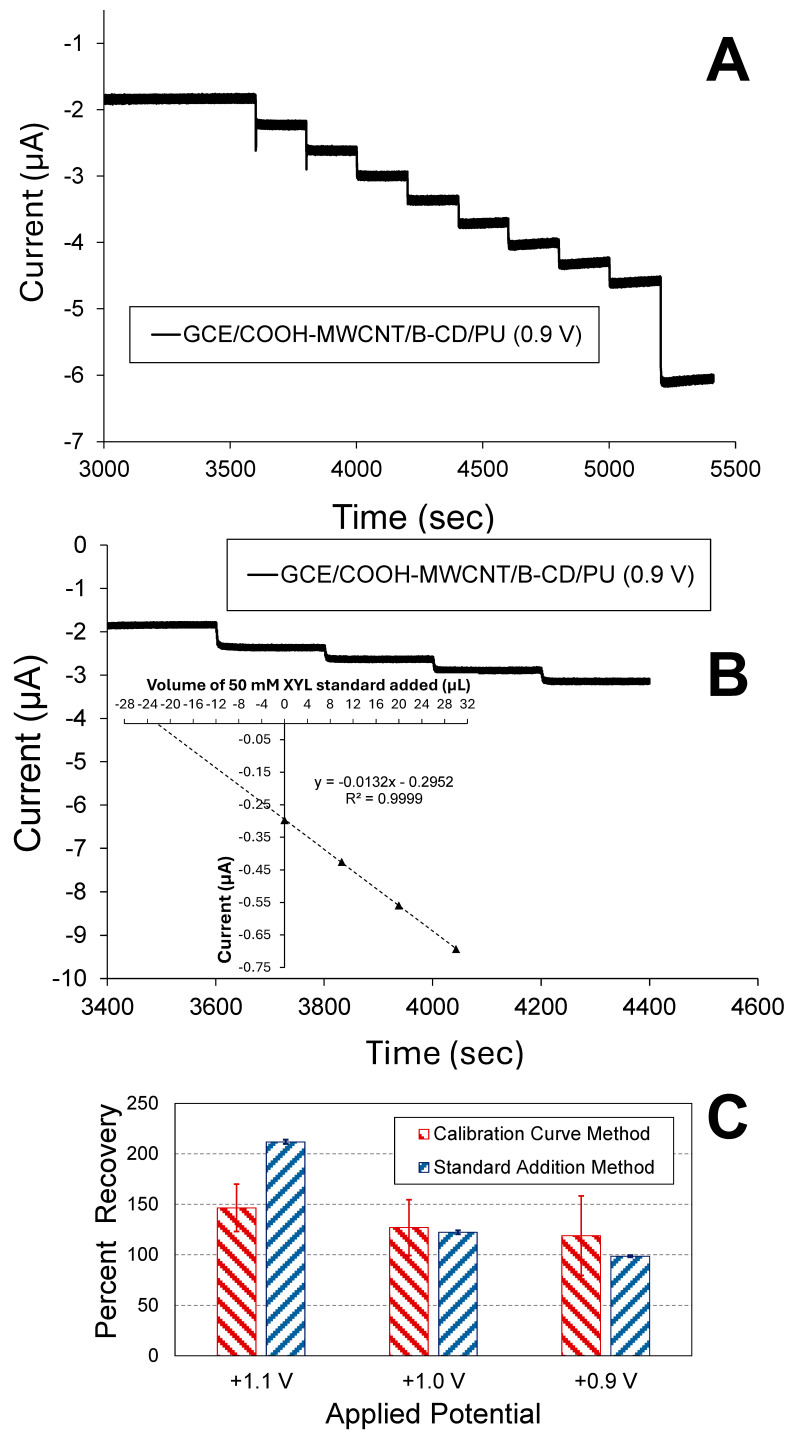
Example amperometric I-t curves using (**A**) calibration curve (successive 10 µL injections of 50 mM XYL standard over a concentration range of 20 to 160 µM) followed by a XYL spike, and (**B**) standard addition methodology, with typical standard addition plot (inset) used to quantify XYL in spiked 150 mM PBS solutions at the fully modified electrode systems (GCE/COOH-MWCNT/β-CD/HPU). (**C**) Bar graph comparison of calculated percent recovery values at different applied potentials using the calibration curve and standard addition method targeting XYL spike concentrations of 260 and 40 µM, respectively (n = 4–6).

To test this hypothesis, comparative experiments were designed where both CC analysis using conditioned sensors and SA analysis of low-concentration XYL-spiked samples of PBS solutions were tested at different applied potentials (+0.9, +1.0, +1.1 V). In this set of experiments, the lower applied potential allowed for the partial control of XYL oxidation by exhibiting smaller charging currents that allow XYL injections to start sooner combined with SA analysis to compensate for matrix effects. This requires fewer total XYL injections that lower the overall XYL concentration and prevents subsequent fouling experienced by the sensor. If the hypothesis is correct, the percent recoveries for both the CC and SA analyses should be improved by lowering the applied potential and utilizing the SA analysis. Figure 6A represents the I-t calibration and spike measurement with conditioned electrodes and its corresponding CC (inset). Figure 6B shows an example of an SA analysis with the results of both experiments compared in the bar graph (Figure 6C). The hypothesis was clearly supported in that the lower applied potentials delivered better percent recoveries, with the SA analysis at a nearly 100% recovery with a notably significant lower variability.

With the optimized sensor fabrication coupled with its optimal quantitative methodology and application, XYL was successfully detected and quantified in a number of common beverages including Coke, Diet Coke, vodka, tequila, gin, and rum. As with the soda beverages, the alcoholic beverages were first tested for potential unidentified interferents by running amperometry during the injections of the liquids without dilution (Supporting Information, Figure S14). From these results, it is notable that the injection of the alcoholic beverages as received in the bottle yielded very little current responses even though they were injected at a significantly higher volume (500 µL) than was XYL. In order to apply the sensors to actual beverages, mimics of strong cocktails or mixed drinks were prepared by mixing each liquor with an equal part of PBS prior to spiking that solution with XYL at ~18 mM. Critically, only 20 µL of these mixed drinks was transferred to PBS for the amperometric XYL testing. This small volume was important because it suggests that the sensors were sensitive enough to detect XYL at micromolar concentrations and allowed for the analysis of drink residue (i.e., small amounts of leftover beverage after consumption that may be further diluted by melting ice, for example). As summarized in Table 1, XYL was detected using an optimized modified electrode sensor at an applied potential of +0.9 V with a standard addition analysis. Most of the analyses resulted in excellent percent recoveries of approximately 100% with low variability. Examples of the SA I-t curves and corresponding plots are provided in Supporting Information (Figures S15–S17). While studies of XYL electrochemical detection in beverages are limited in number, a table of comparison of the various works is included in Supporting Information (Table S1). We note, however, that direct comparison is rather difficult as the studies use different components, techniques, and applications that render specific advantages and disadvantages to each system.

## 4. Conclusions

A portable sensor capable of effectively screening for XYL in beverage samples and/or from the residue of a consumed beverages would serve as an important tool for preventing and/or investigating sexual assaults and other drug-related cases. XYL, however, is well-known to foul electrodes under oxidizing potentials, posing a challenge for designing an effective electrochemical sensor. While materials such as CNTs, β-CD, and semi-permeable PU blends can enhance selectivity and sensitivity, they can also introduce new variables such as increased charging currents. The sensor scheme developed in this study strives to achieve a balance of such critical variables: fouling resistance, signal-to-noise ratios, and ease of fabrication and use by non-experts. The results from these robust sensors suggest they could be effective for indicating XYL presence using only small sample volumes of a beverage suspected to be spiked with XYL or directly in the beverage sample as well [38]. This type of versatility is critical for presumptive screening tests of this nature, particularly when XYL can be present in beverages in vastly different concentrations or combined with other substances [38]. This successful proof-of-concept study can serve as the premise for testing this system further, including the incorporation of other semi-permeable layers [39,40,41] that may further eliminate potential interferents and ultimately decrease the number of false positives from the system. Overall, the study supports a XYL sensing platform comprised of common, low-cost components that employs simple amperometry (applied potential) that can both be miniaturized and mass produced for use by non-experts onsite.

## Figures and Tables

**Table 1:** Quantitative analysis of XYL in beverages with standard addition (+0.9 V).

Beverage	Volume Sampled (µL)	Dilution in 150 mM PBS (mL)	Spike [XYL] ^b^ (µM)	Trials (n)	Avg [XYL] Measured (µM)	Avg % Recovery ^c^
Coke^TM^	50	25	36.3	6	36.8 (±5.8)	101%
Diet Coke^TM^	20	25	14.5	5	14.3 (±1.2)	98%
Vodka Mix ^a^	20	25	14.5	6	15.4 (±2.5)	106%
Tequila Mix ^a^	20	25	14.5	6	14.5 (±1.0)	100%
Gin Mix^a^	20	25	14.5	4	14.9 (±0.9)	103%
Rum Mix^a^	20	25	14.5	7	15.2 (±2.2)	105%

Notes: Coke and Diet Coke were used without dilution and spiked with XYL (18.15 mM). ^a^ Alcoholic beverages were mimicked by mixing as-received liquor 1:1 with PBS (150 mM) and spiked with XYL (18.15 mM). Standard addition performed with a 50 mM XYL standard solution in PBS (150 mM). ^b^ Target spikes [XYL] were 36.6 µM ≈ 8 ppm and 14.5 µM ≈ 3 ppm. ^c^ Uncertainty represents standard error.

## Data Availability

The data that support the findings of this study are available from the corresponding author upon reasonable request.

## Supplementary Materials

The following supporting information can be downloaded at: https://www.mdpi.com/article/10.3390/mi15111340/s1, Figures S1–S4: I-t curves and corresponding calibration curves for modified electrodes with different nanomaterials, capping layers, PU blends, and PU layer thickness; Figure S5: I-t curves and corresponding calibration curves for different PU blends at fully modified electrodes; Figure S6: I-t curves and current tracking of XYL injections at fully modified electrodes with different sonication applications; Figure S7: typical XYL voltammetry at bare GCEs; Figure S8: signal/current potential dependence; Figure S9: interferent testing (diet soda); Figures S10–S13: I-t curve and calibration curves for XYL calibrations in simulated soda, simulated diet soda, Coke, and Diet Coke; Figure S14: interferent testing (alcoholic beverages); Figures S15–S17: I-t curves and corresponding plots for the standard addition analysis of different beverages. Table S1: comparison of XYL sensor reports from the literature.
