# Supplementary material for: An Amperometric Sensor with Anti-Fouling Properties for Indicating Xylazine Adulterant in Beverages
Source: Micromachines (Basel). 2024 Oct 31;15(11):1340. doi: 10.3390/mi15111340 (PMC11596286; doi:10.3390/mi15111340)
Supplement: Supplementary file 1 [file micromachines-15-01340-s001.zip › micromachines-3286112-supplementary.pdf]

Supporting Information: An Amperometric Sensor with Anti-fouling Properties for Indicating Xylazine Adulterant in Beverages

Arielle Vinnikov†, Charlie W. Sheppard†, Ann H. Wemple, Joyce E. Stern, and Michael C. Leopold*

**Department of Chemistry, Gottwald Center for the Sciences, University of Richmond, Richmond, Virginia 23173, United States (Corresponding Author)*

Table of Contents:

- Amperometric I-t curves and corresponding calibration curves for modified electrodes featuring different types of nanomaterials (**Fig. S1**).
- Amperometric I-t curves of modified electrodes comparing different capping layers of Nafion and PU (75:25) (**Fig. S2**).
- Amperometric I-t curves and current tracking during XYL injections at bare GCEs modified with different blends of PU (different ratios of HPU to TPU) (**Fig. S3**).
- Amperometric I-t curves and corresponding calibration curves during XYL injections at fully modified GCEs (GCE/COOH-MWCNT + β-CD/PU with different thicknesses of PU (75:25) (**Fig. S4**).
- Amperometric I-t curves during XYL injections at fully modified GCEs (GCE/COOH-MWCNT + β-CD/PU with different blends HPU to TPU (i.e., different ratios) (**Fig. S5**).
- Amperometric I-t curves and current tracking during XYL injections at fully modified GCE where the MWCNTs and β-CD were either sonicated together and deposited as a layer or deposited as layers separately (**Fig, S6**).
- Typical cyclic voltammogram of XYL at a bare GCE **(Fig. S7).**
- Measured currents for fully modified electrodes held at different potentials during XYL injections (corresponding to the I-t curve comparison of Figure 2C (**Fig. S8**).
- Interferent testing with amperometric I-t curves and selectivity coefficient graph for diet soda (**Fig. S9**).
- Representative I-t curve and calibration curves for XYL calibrations in simulated soda, simulated diet soda, Coke, and Diet-Coke (**Figs. S10 to S13**).
- Example amperometric I-t curve testing for interferents in the different alcoholic beverages (**Fig. S14**).
- Representative examples of I-t curves and corresponding plots for the standard addition analysis of different beverages (**Figs S15 to S17**).
- **Table S1**: comparison of XYL sensor reports from literature.

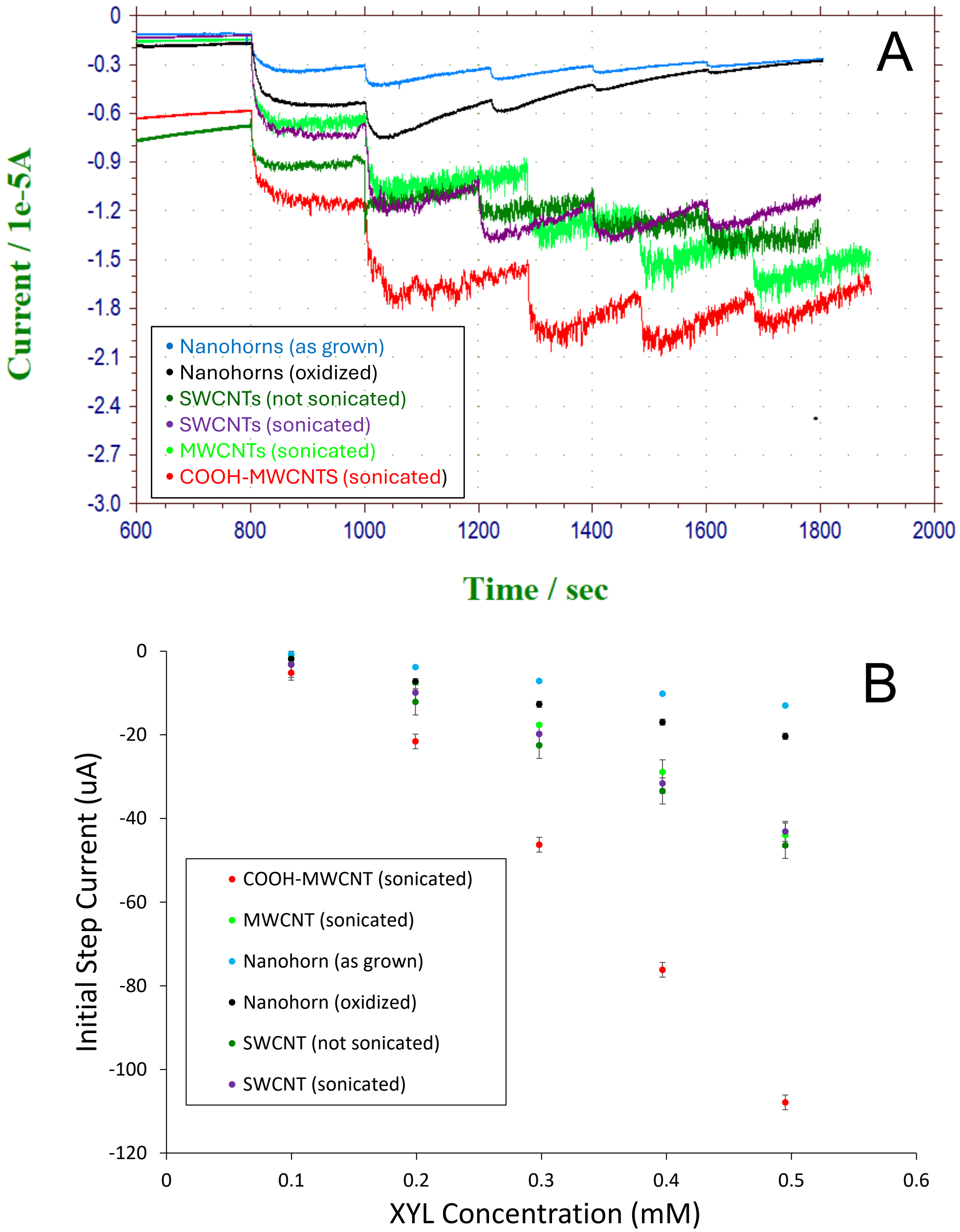

Figure S1. **(A)** Representative amperometric I-t curves during five successive injections of 0.1 mM XYL at GCE electrodes modified with various nanomaterials (sonicated with β-CD and deposited (7 µL) and capped with a Nafion layer (7 µL); **(B)** initial step current tracking with various incorporated NMs (n=3).

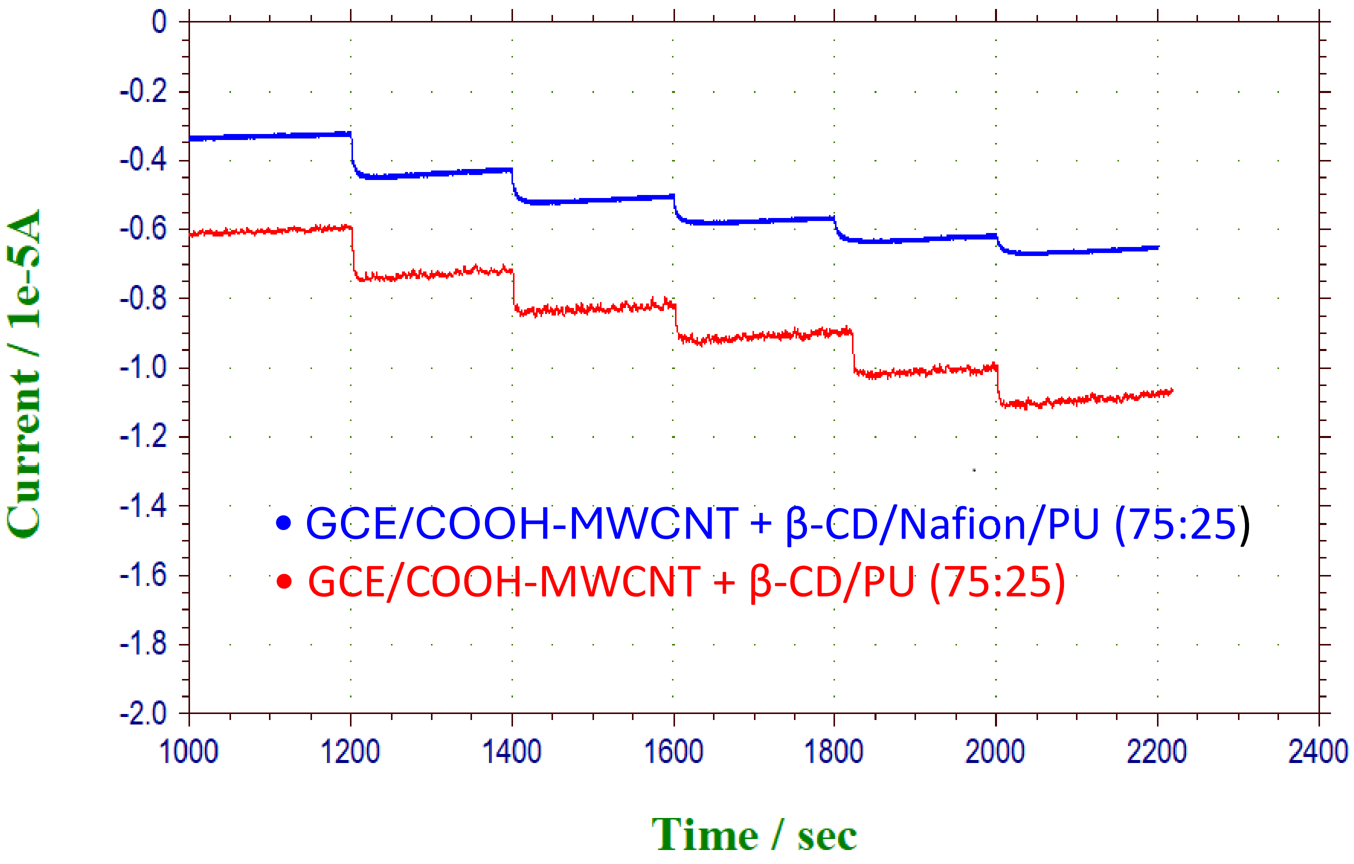

Figure S2. Representative amperometric I-t curves during five successive injections of 0.1 mM XYL at GCE electrodes modified COOH-MWCNTs with β-CD and capped with either Nafion and an outer PU blended layer or only a PU-blended layer. More defined and regular stepping was consistently observed on systems without the Nafion layer and sonication technique/application did not affect this trend.

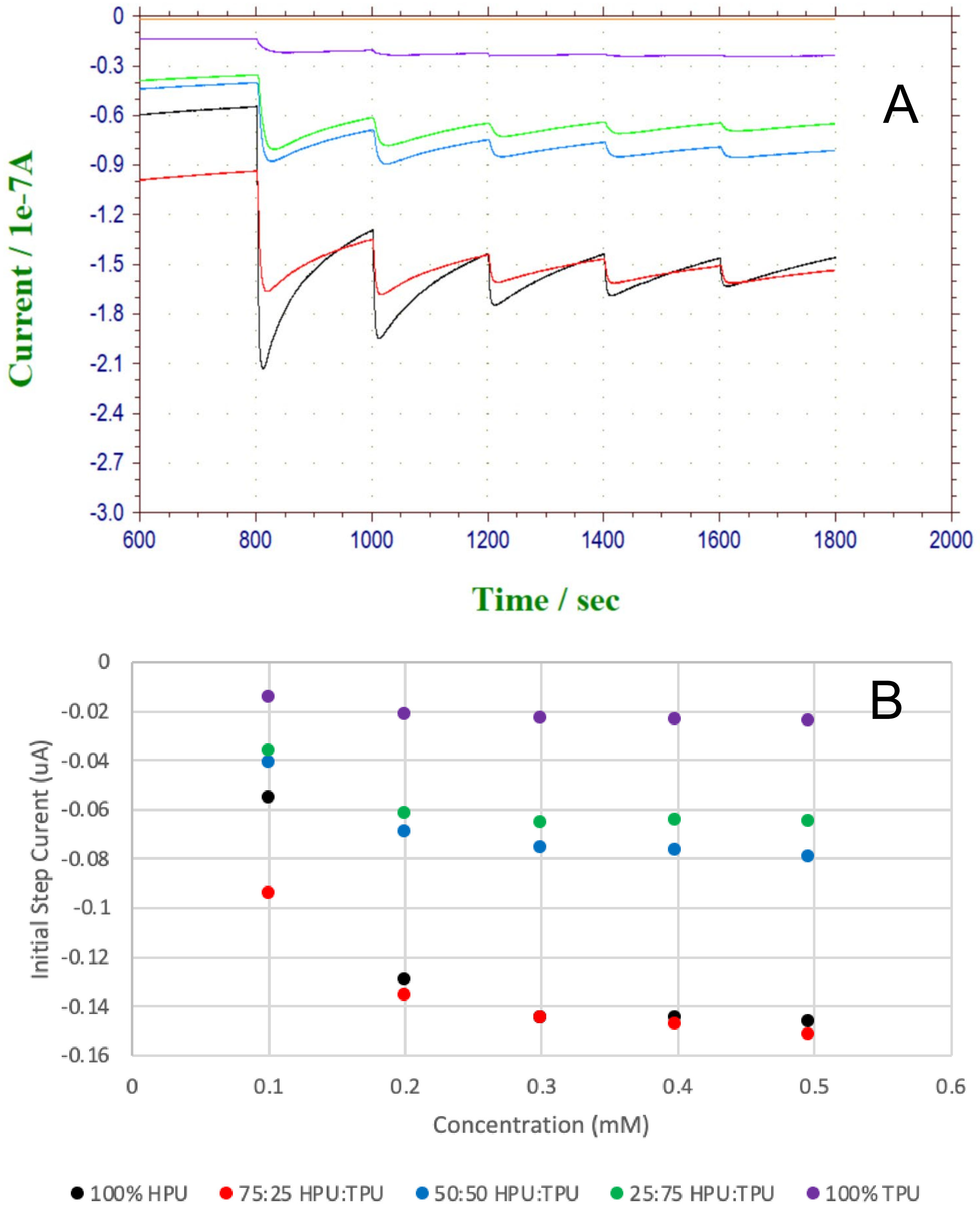

Figure S3. **(A)** Representative amperometric I-t curves and **(B)** corresponding current tracking example during five successive injections of 0.1 mM XYL at unmodified (bare) GCEs modified with only different blends of PU (i.e., different ratios of HPU to TPU).

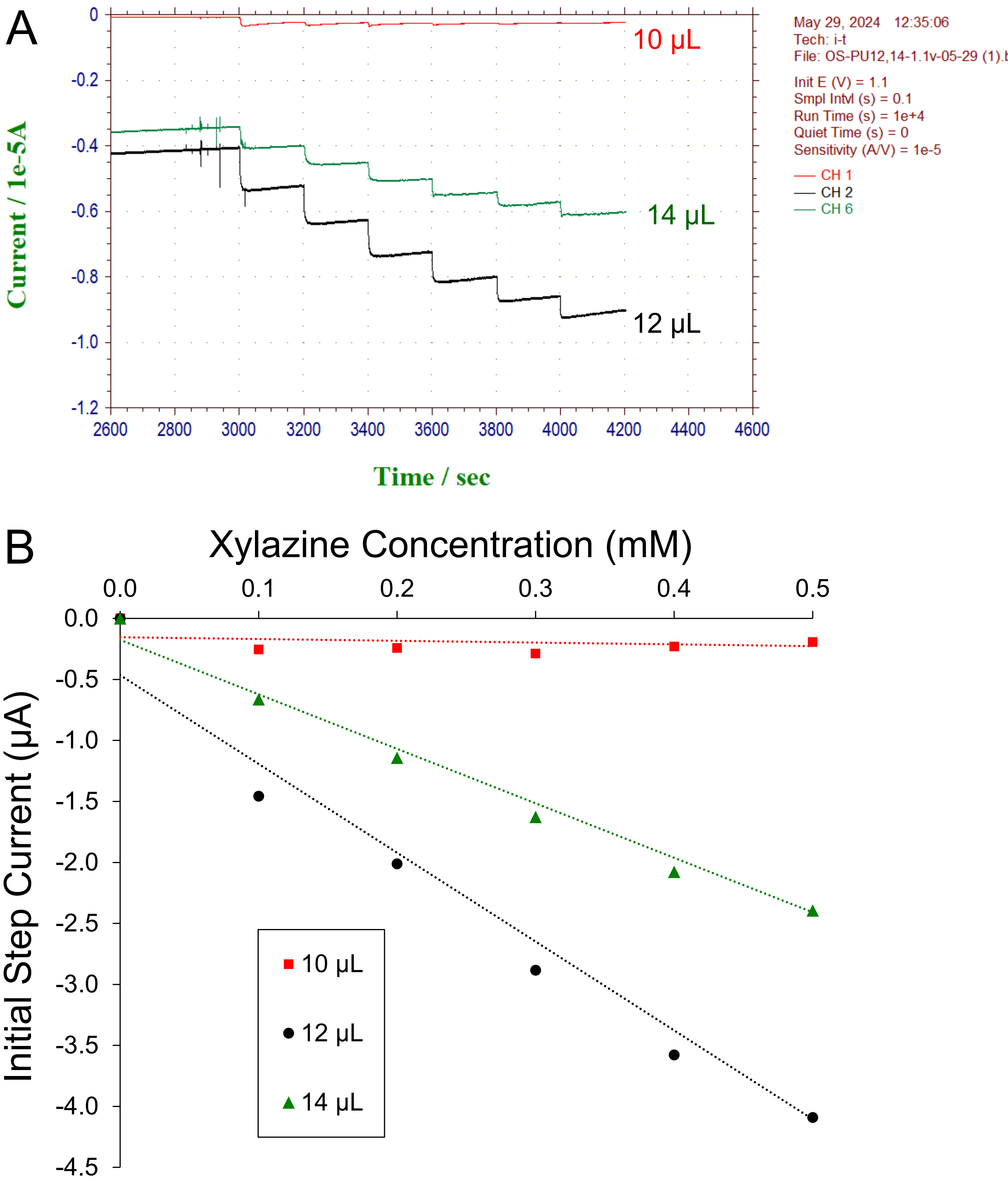

Figure S4. **(A)** Representative amperometric I-t curves and **(B)** corresponding current tracking example during five successive injections of 0.1 mM XYL at fully modified electrodes (GCE/COOH-MWCNT/β-CD/PU (75:25) where PU deposition volume (thickness of PU layer) is varied.

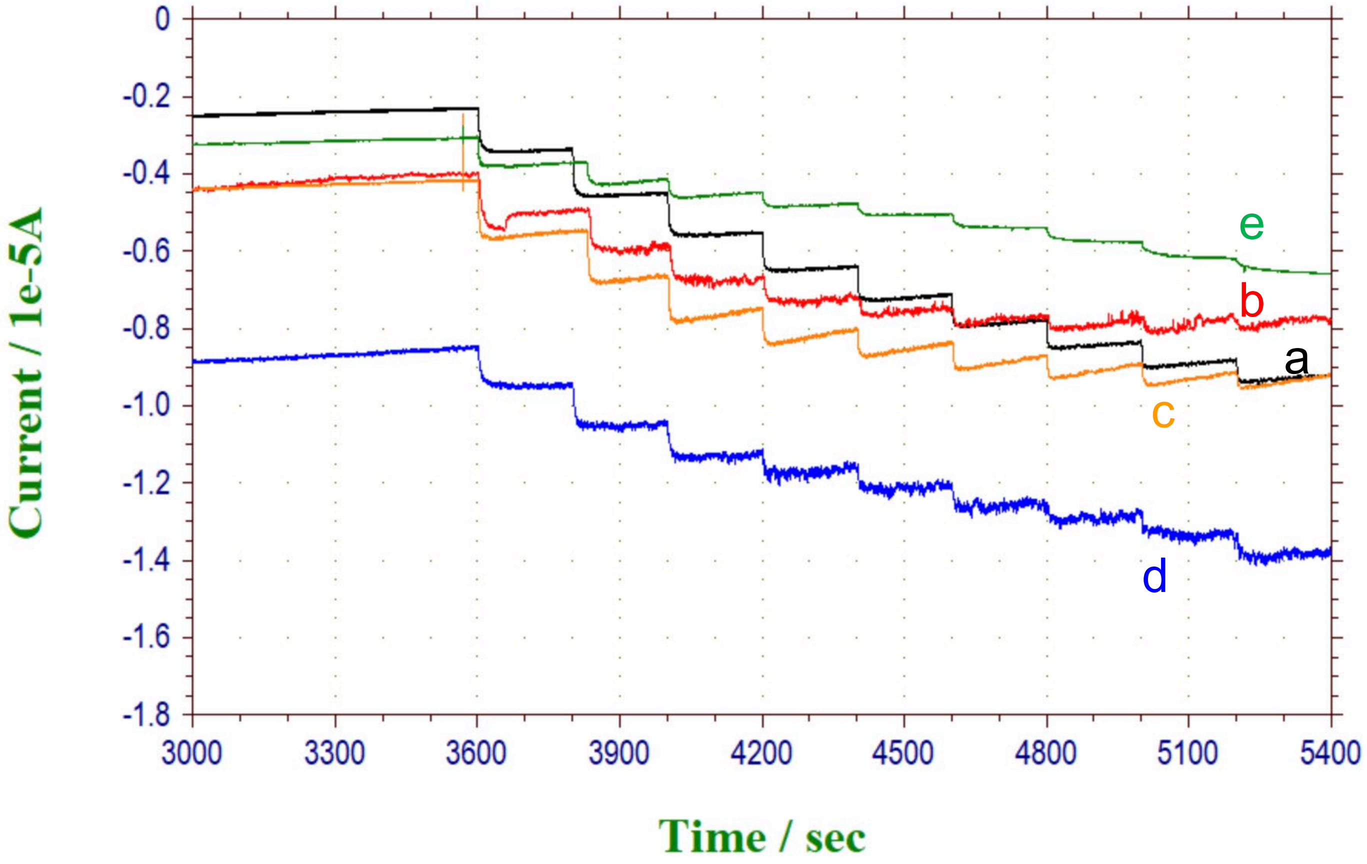

Figure S5. Representative amperometric I-t curves during successive injections of 0.1 mM XYL at fully modified GCE (GCE/COOH-MWCNT/PU) featuring different composition ratios (i.e., blends) of HPU to TPU: (a) 100:0; (b) 75:25; (c) 50:50; (d) 25:75; (e) 0:100.

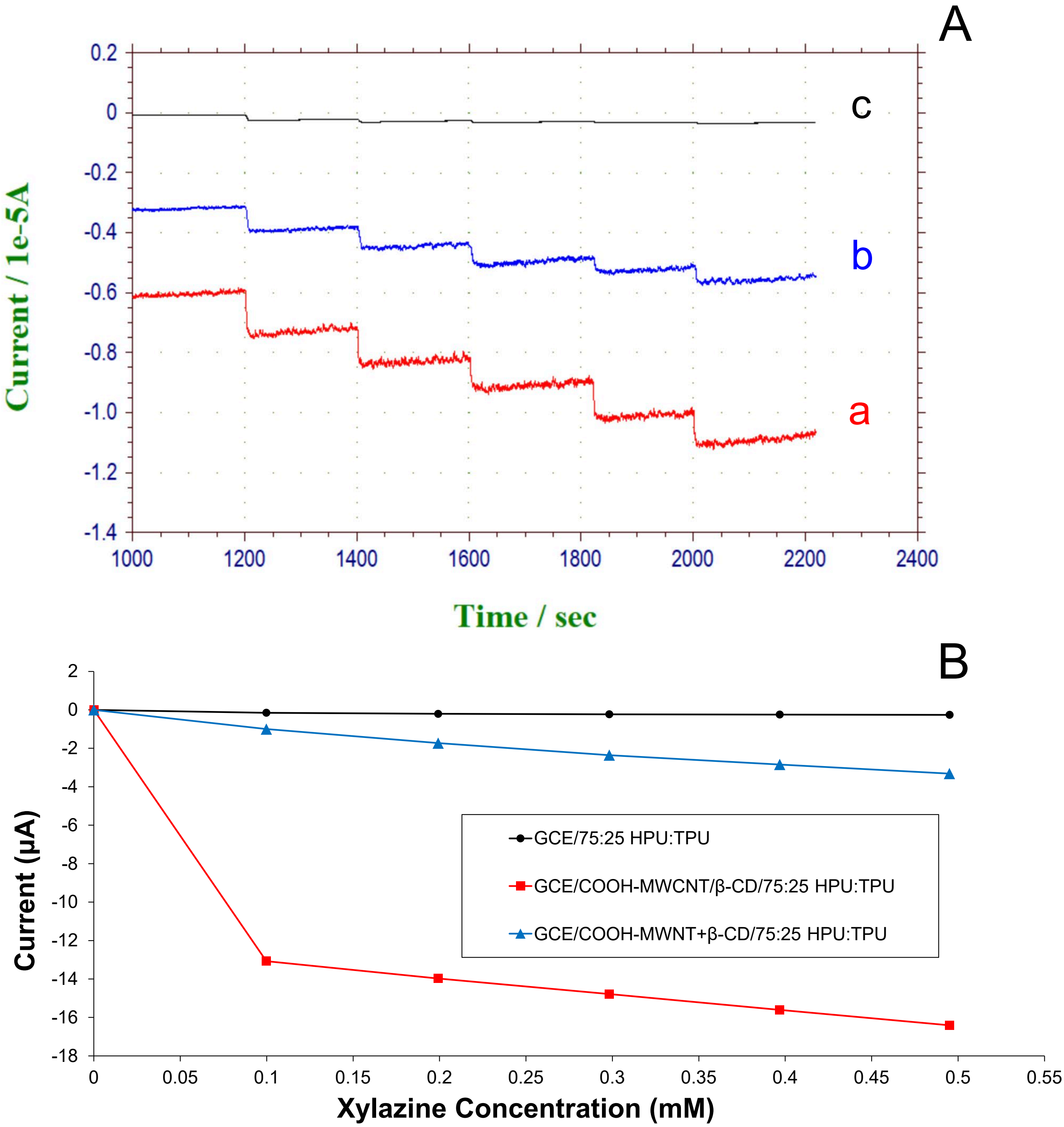

Figure S6. **(A)** Representative amperometric I-t curves and **(B)** corresponding tracking of current as a function of XYL concentration during five successive injections of 0.1 mM XYL at GCE electrodes modified with COOH-MWCNTs and β-CD and capped with PU (75:25) where the MWCNT and β-CD were **(a)** layered separately after sonication, **(b)** sonicated as a mixture and layered together, or **(c)** not incorporated into the film (i.e., GCE modified with only PU (75:25)).

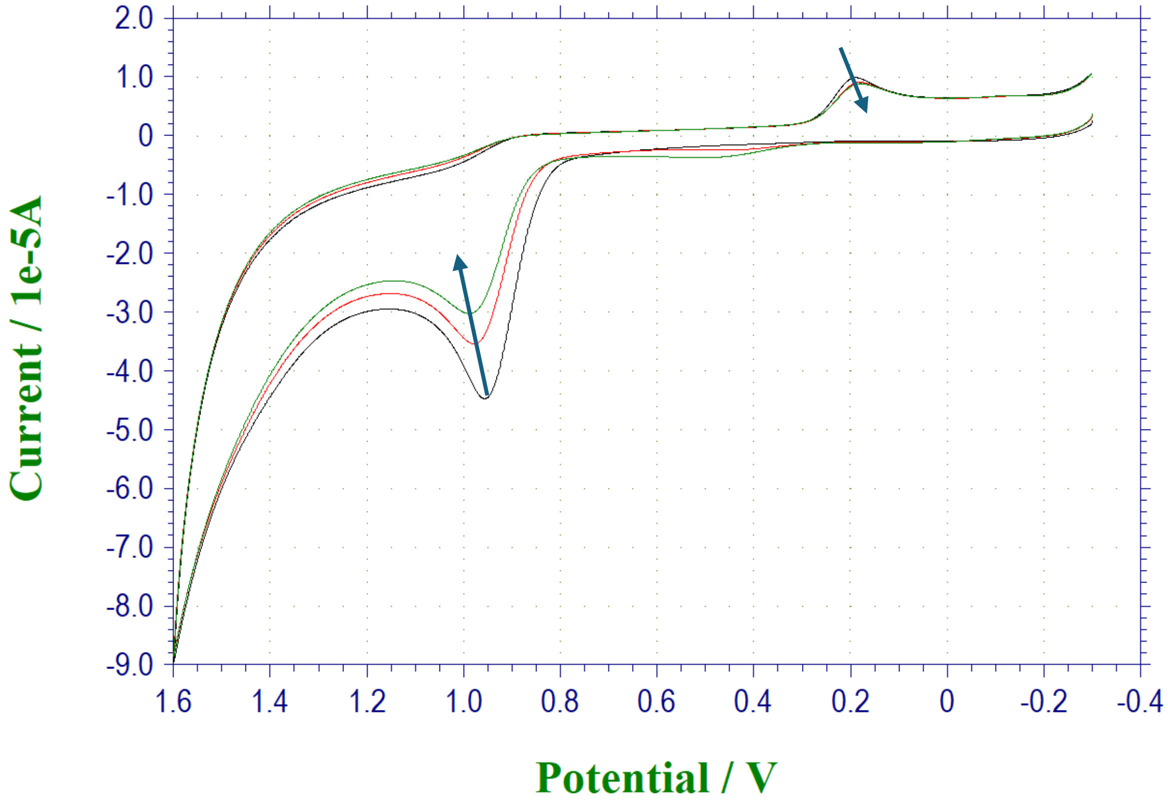

Figure S7. Typical CV of 1 mM XYL in 150 mM PBS (pH = 7) showing three full scans of XYL at a bare/unmodified GCE with peaks showing XYL oxidation at ~ 0.95 V and the reduction of that oxidation product at ~0.2 V (vs. Ag/AgCl, satrd. KCl) including evidence of electrode fouling as oxidation peaks diminish and shift more positive with each scan.

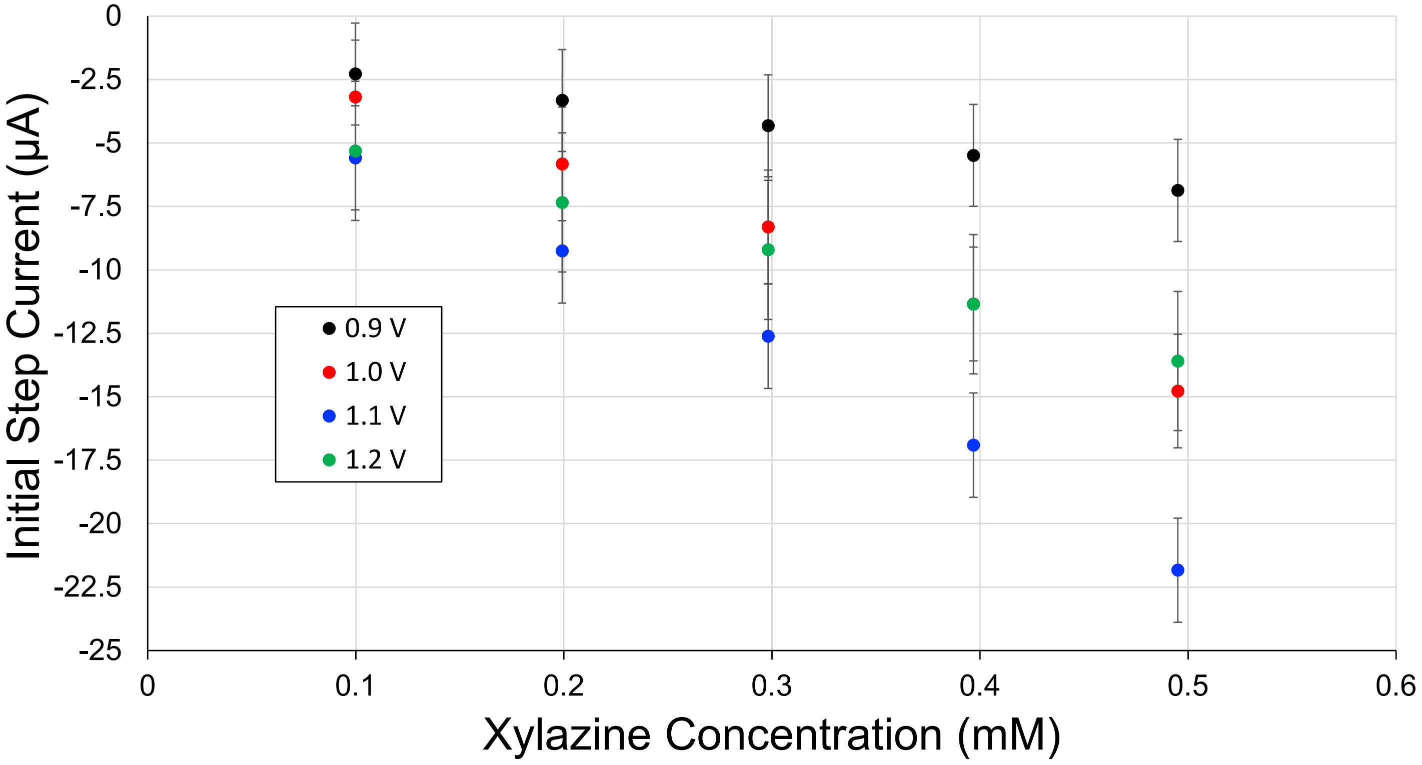

Figure S8. Example of corresponding current tracking from **Fig. 2C** for fully-modified electrode (GCE/COOH-MWCNT/β-CD/PU(75:25) during injections of 0.1 mM XYL at different applied holding potentials: +0.9, +1.0, +1.1, and +1.2 V.

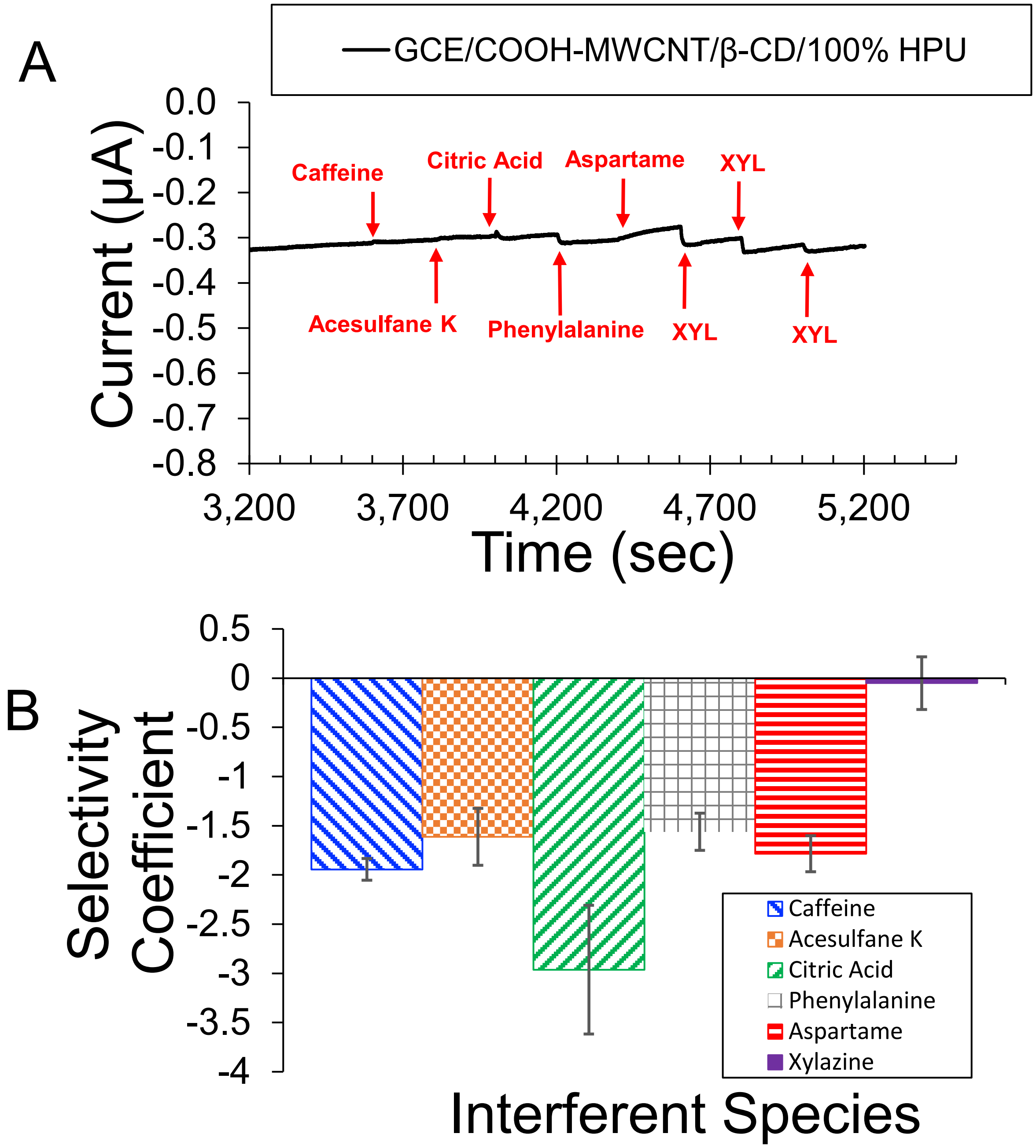

Figure S9. (A) Amperometric I-t curves during XYL injections (50 µL of 50 mM XYL standard) and potential interferent species in **diet soda** including (0.49 mM caffeine, 0.21 mM Acesulfane K, 8.95 mM citric acid, 0.95 mM phenylalanine, 2.21 mM aspertame at a modified electrode; **(B)** corresponding graph of calculated selectivity coefficients for the potential interferent species.

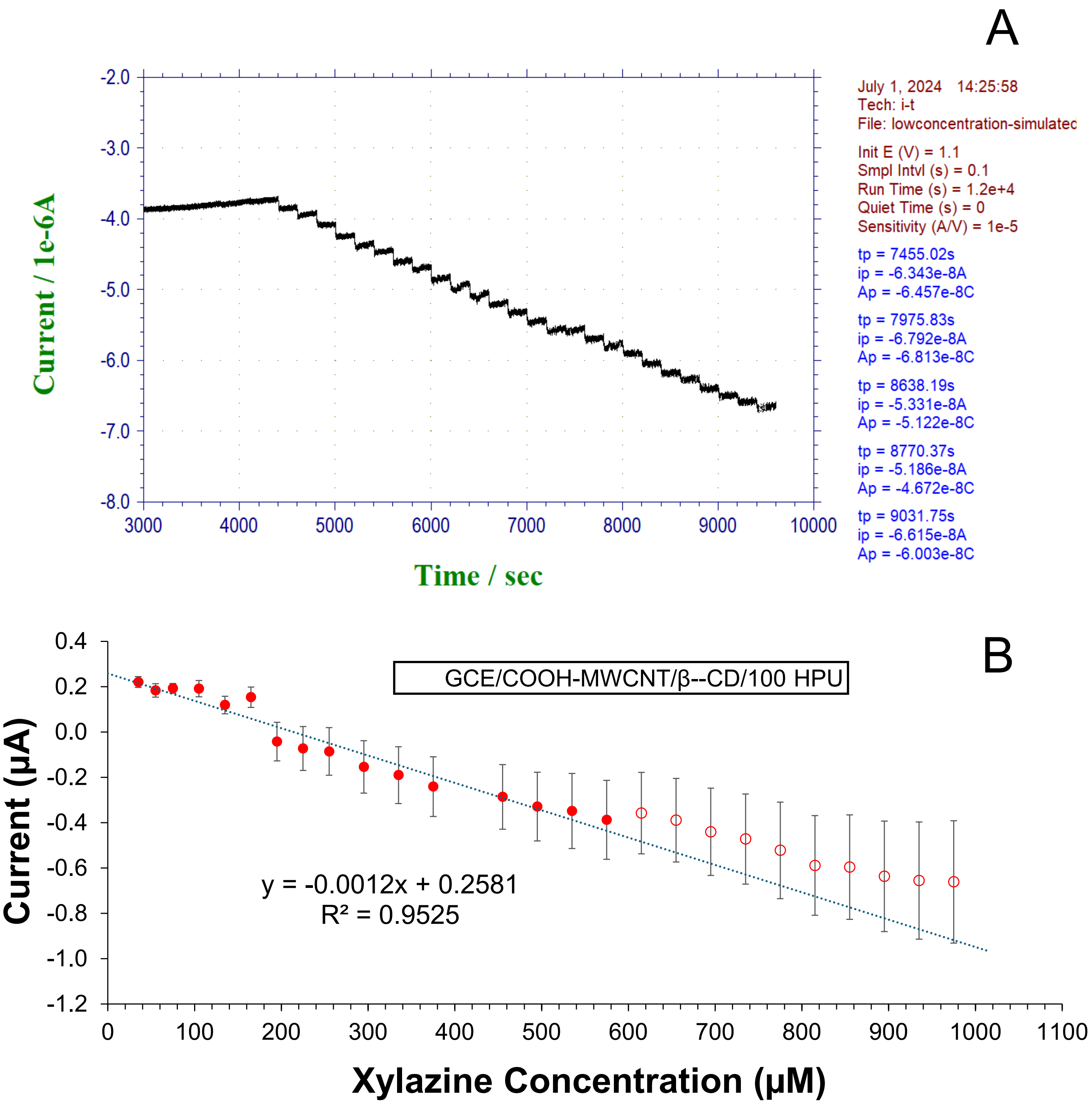

Figure S10. **(A)** Representative amperometric I-t curve example and **(B)** corresponding calibration curve (n=3) for successive injections of 0.1 mM XYL at modified electrodes (GCE/COOH-MWCNT/β-CD / PU (100:0) immersed in a **simulated non-diet soda** solution including the linear range (closed symbols) and dynamic range (open symbols). Note: In some cases, standard error bars are smaller than markers denoting the average.

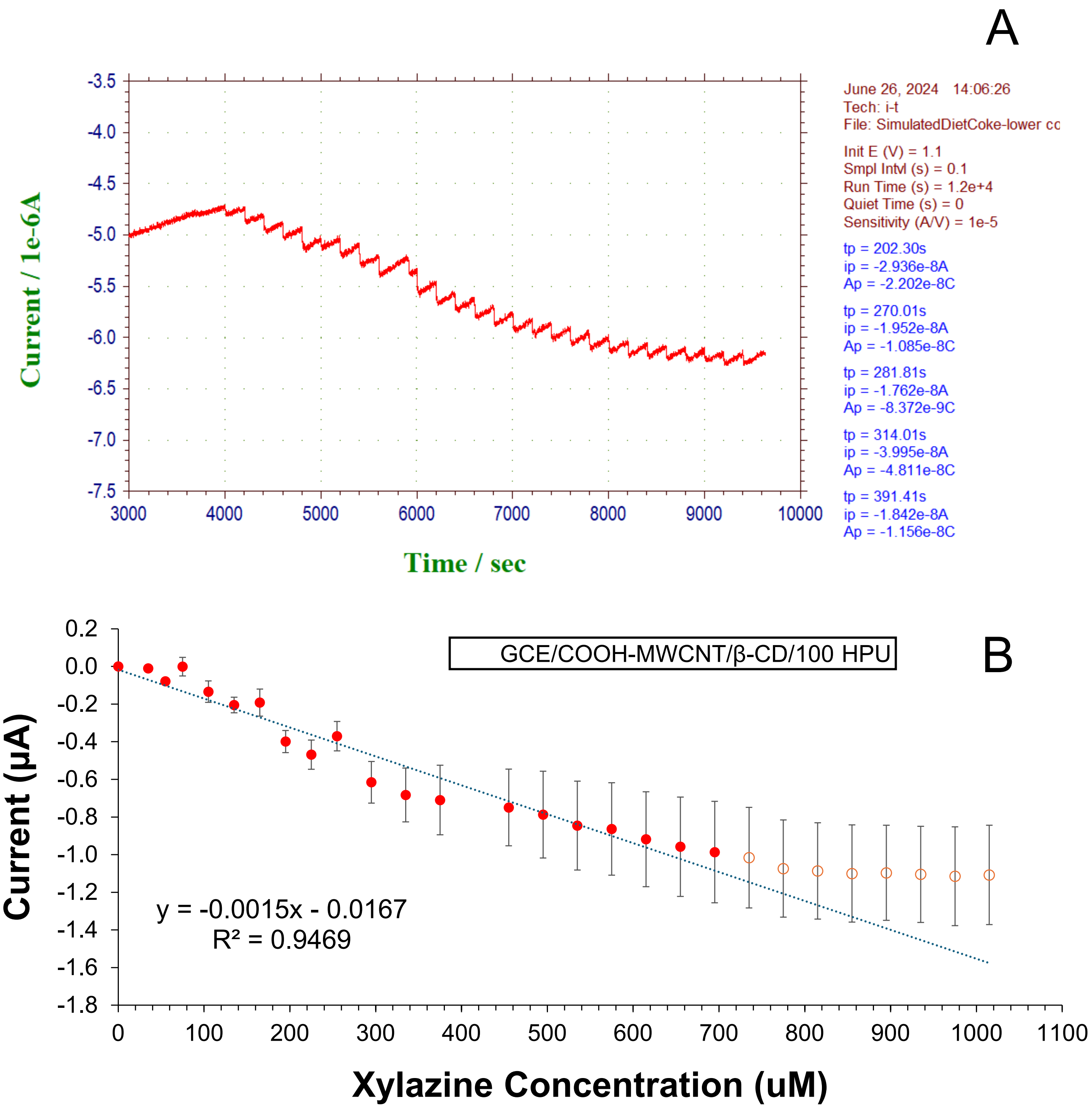

Figure S11. **(A)** Representative amperometric I-t curve example and **(B)** corresponding calibration curve (n=5) for successive injections of 0.1 mM XYL at modified electrodes (GCE/COOH-MWCNT/β-CD / PU (100:0) immersed in **a simulated diet soda** solution including the linear range (closed symbols) and dynamic range (open symbols). Note: In some cases, standard error bars are smaller than markers denoting the average.

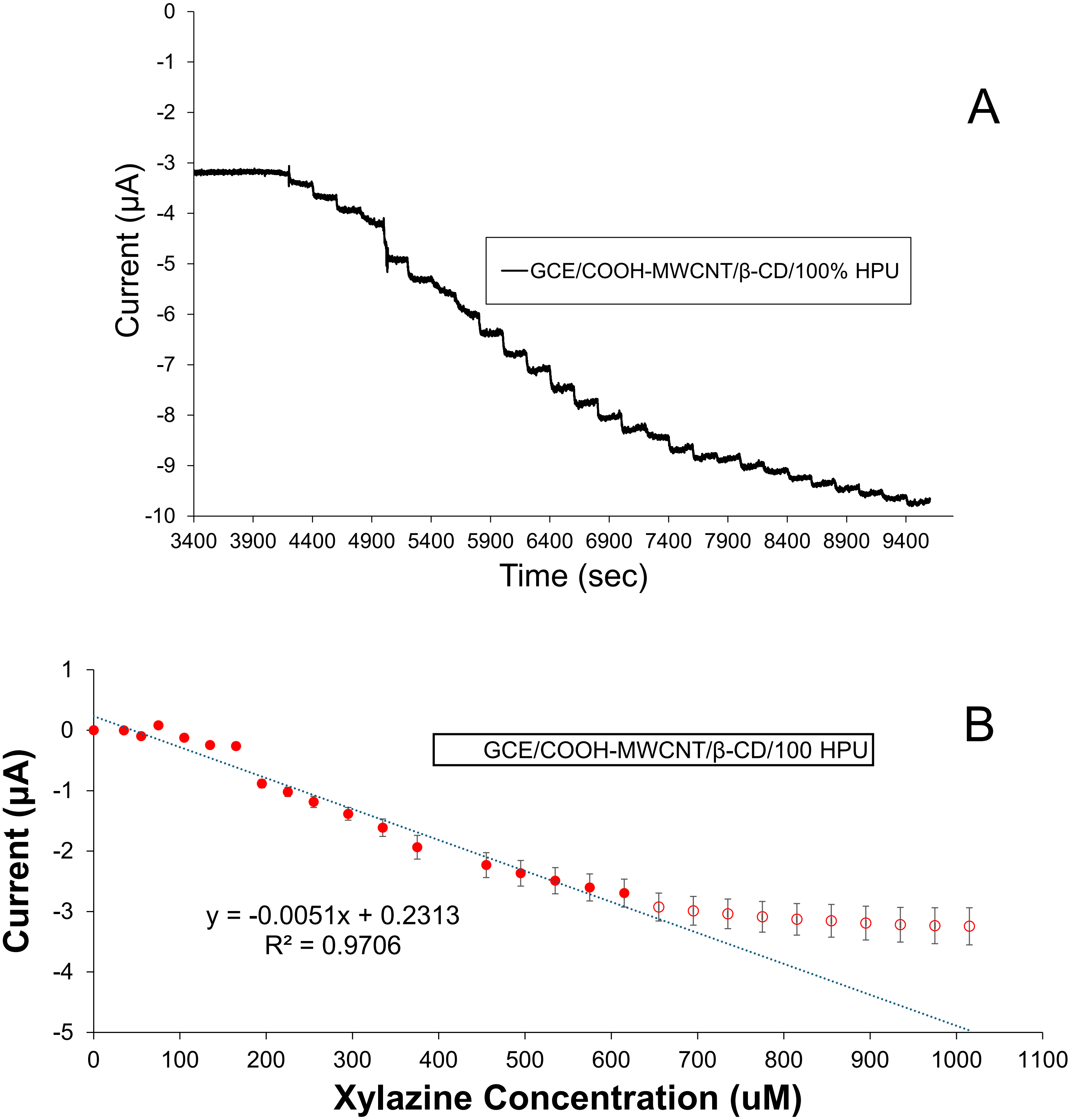

Figure S12. (A) Representative amperometric I-t curve example and **(B)** corresponding calibration curve (n=5) for successive injections of 0.1 mM XYL at modified electrodes (GCE/COOH-MWCNT/β-CD / PU (100:0) immersed in a **soda (i.e., Coke)** solution including the linear range (closed symbols) and dynamic range (open symbols). Note: In some cases, standard error bars are smaller than markers denoting the average.

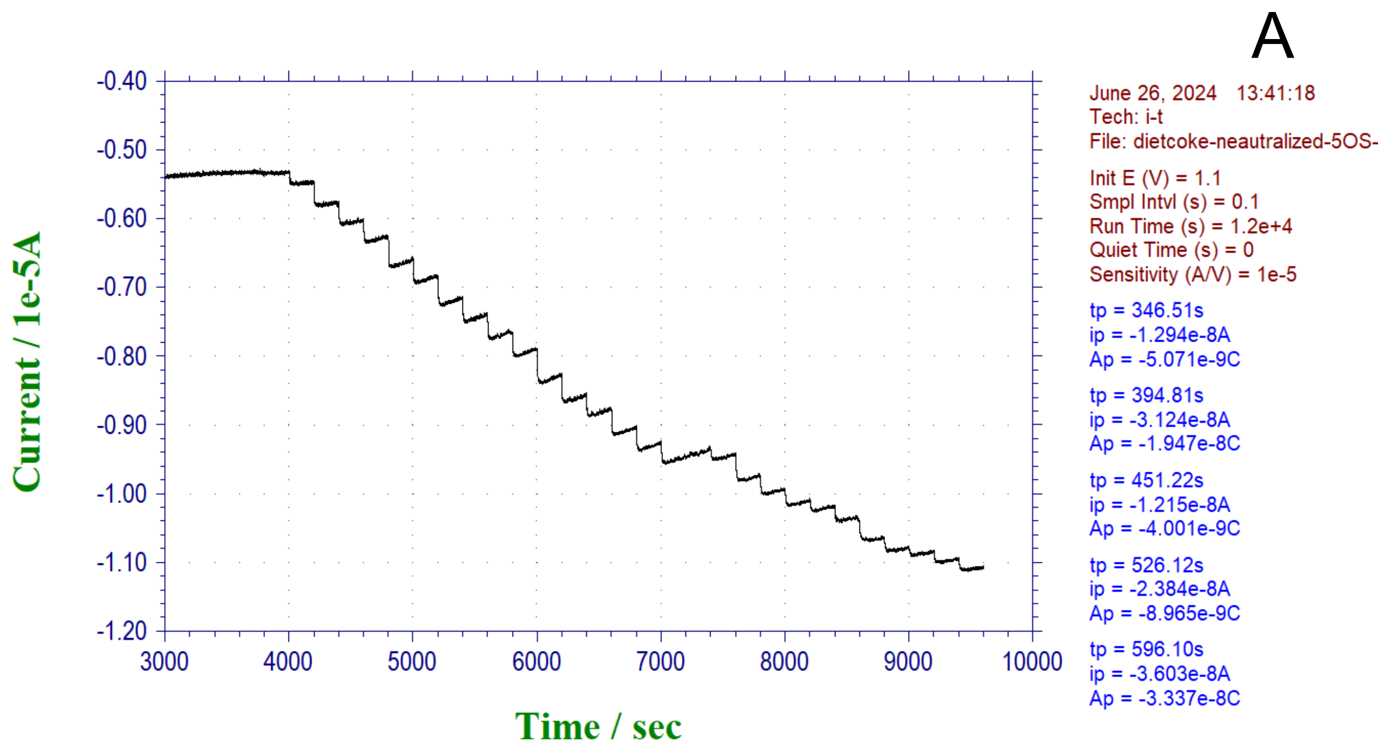

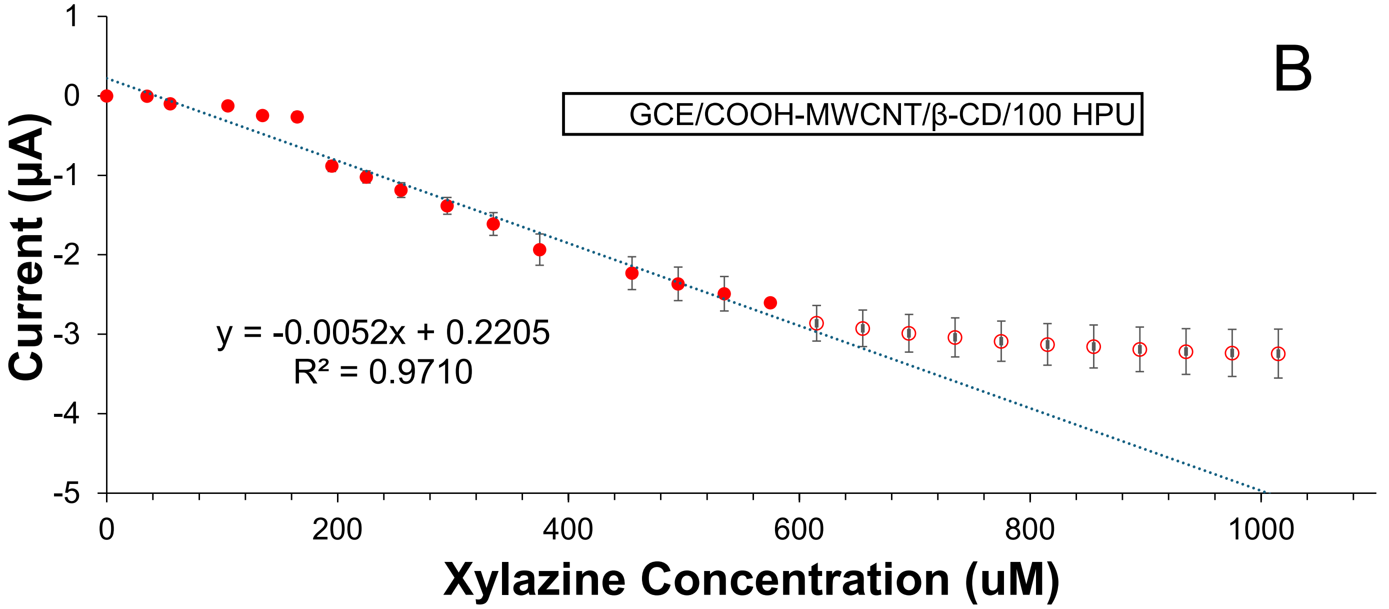

Figure S13. **(A)** Representative amperometric I-t curve example and **(B)** corresponding calibration curve (n=5) for successive injections of 0.1 mM XYL at modified electrodes (GCE/COOH-MWCNT/β-CD / PU (100:0) immersed in a **diet soda (i.e., Diet Coke)** solution including the linear range (closed symbols) and dynamic range (open symbols). Note: In some cases, standard error bars are smaller than markers denoting the average.

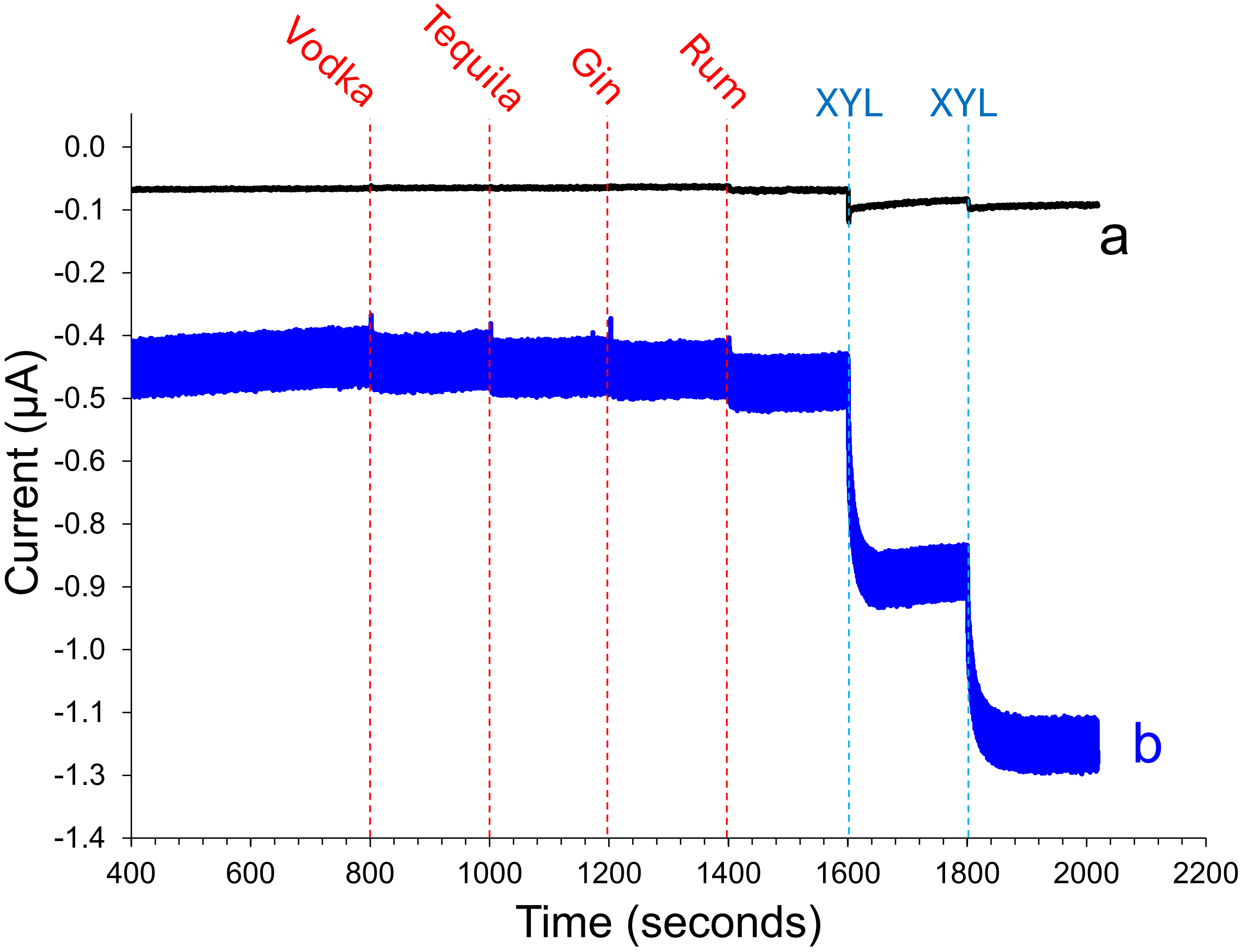

Figure S14. Representative amperometric I-t curves for (a) bare/unmodified GCE versus (b) fully modified GCE (GCE/COOH-MWCNT/β-CD/PU) during successive 500 µL injections of potential interferents (alcoholic beverages) including vodka (800 sec), tequilia (1000 sec), gin (1200 sec), and rum (1400 sec) as well as XYL standards (50 µL of 50 mM XYL at 1600 and 1800 sec) into 25 mL of 150 mM PBS.

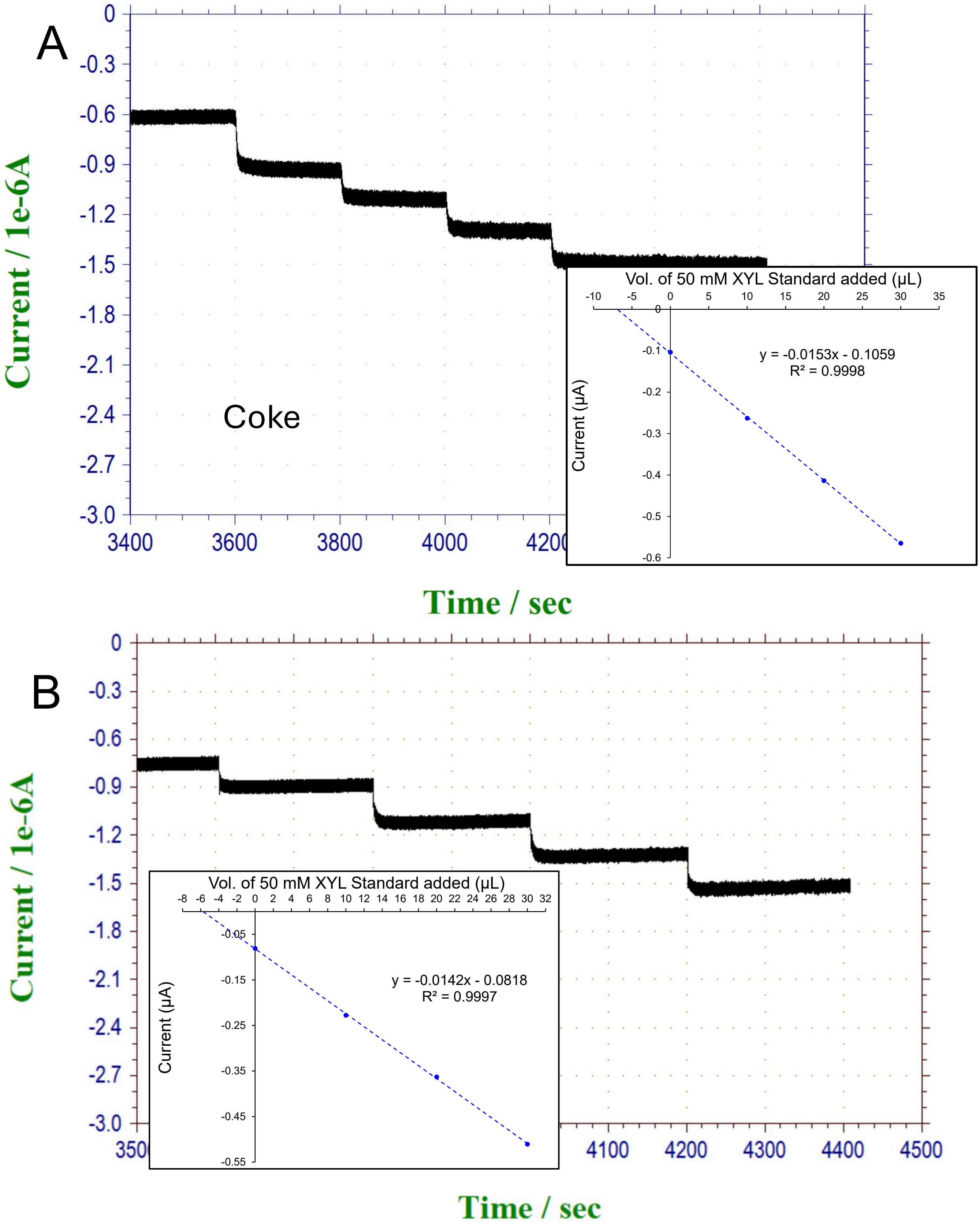

Figure S15. Representative amperometric I-t curve and typical standard addition plot (**inset**) for XYL analysis in (**A**) gin and (**B**) rum.

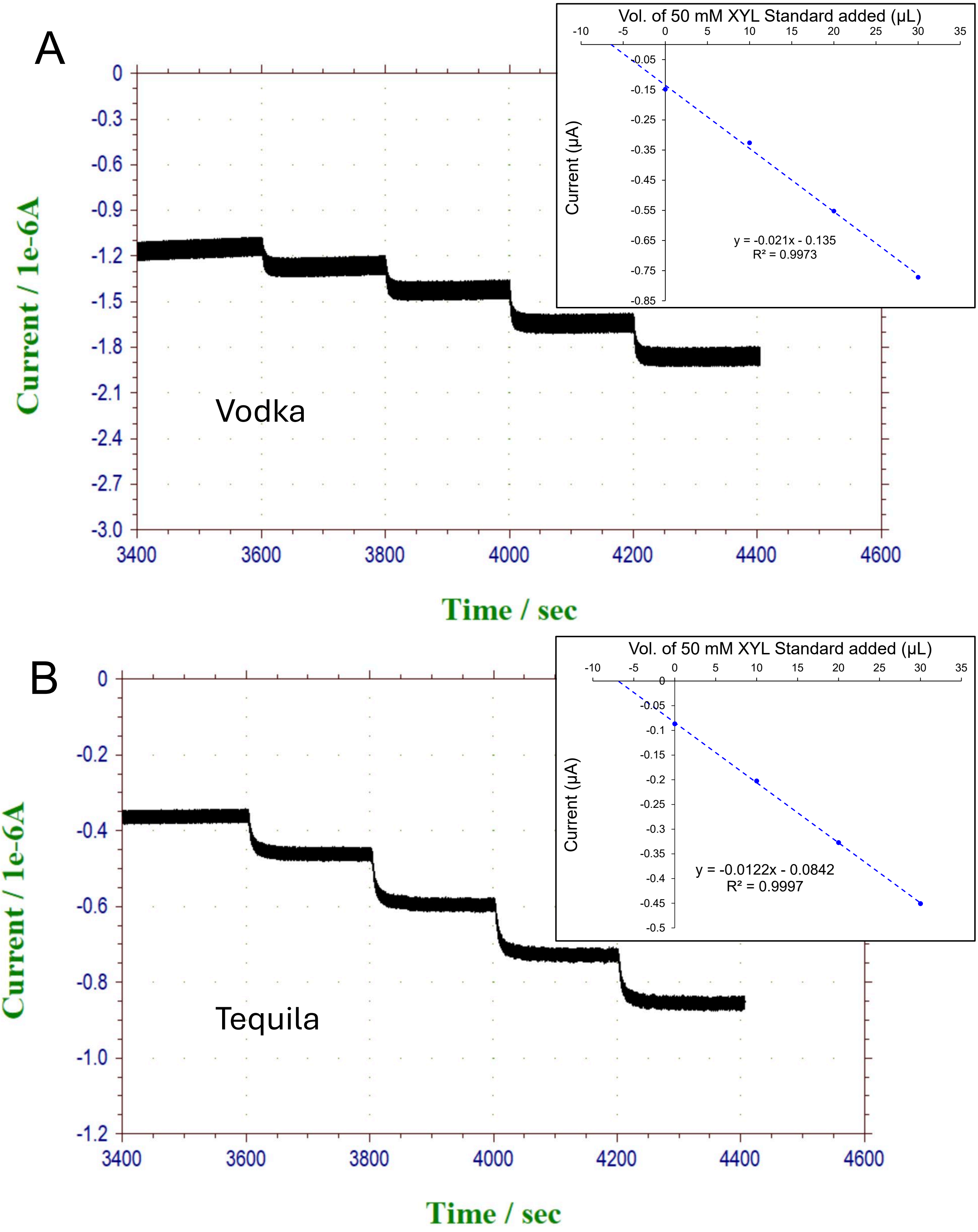

Figure S16. Representative amperometric I-t curve and typical standard addition plot (**inset**) for XYL analysis in (**A**) vodka and (**B**) tequila.

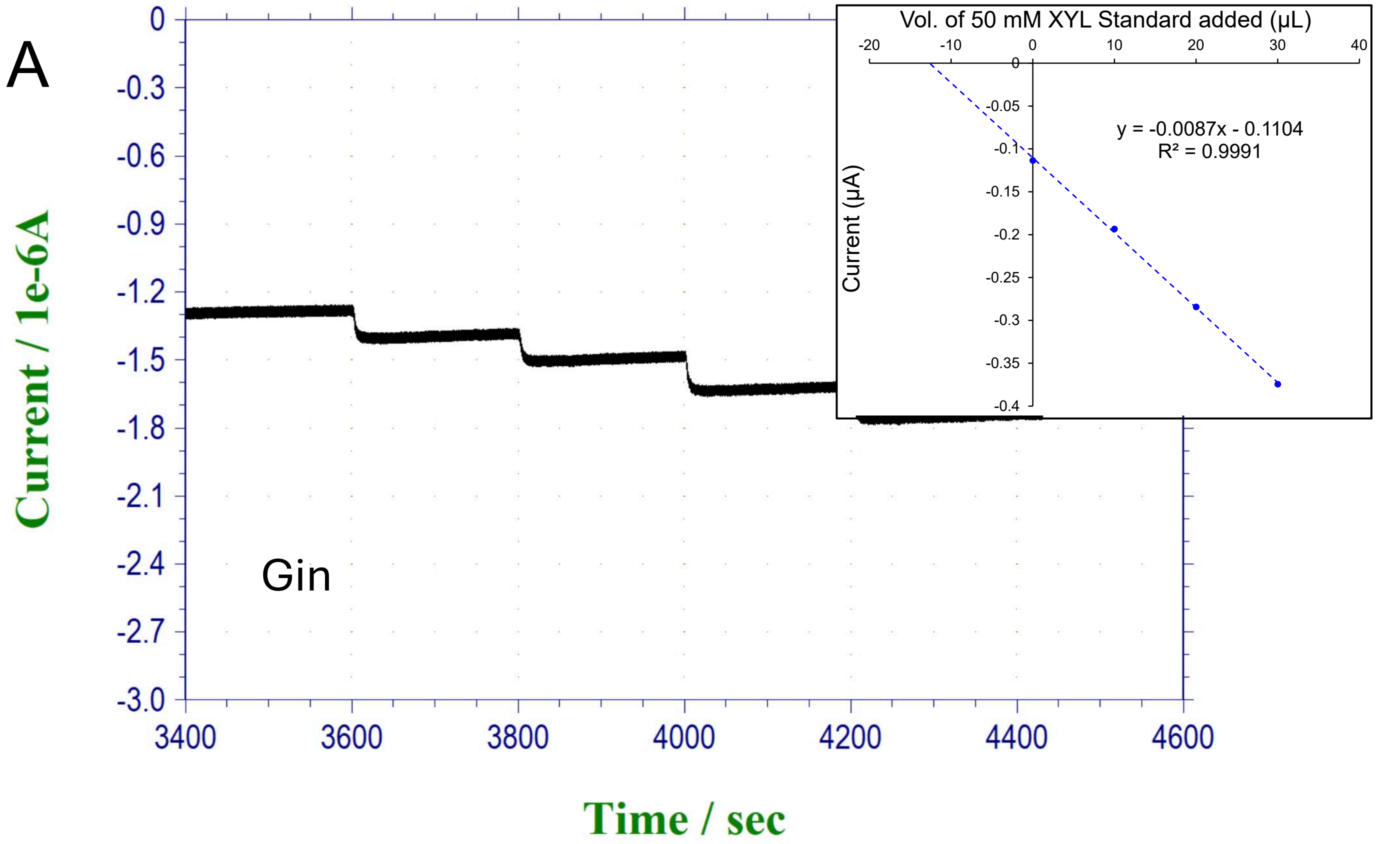

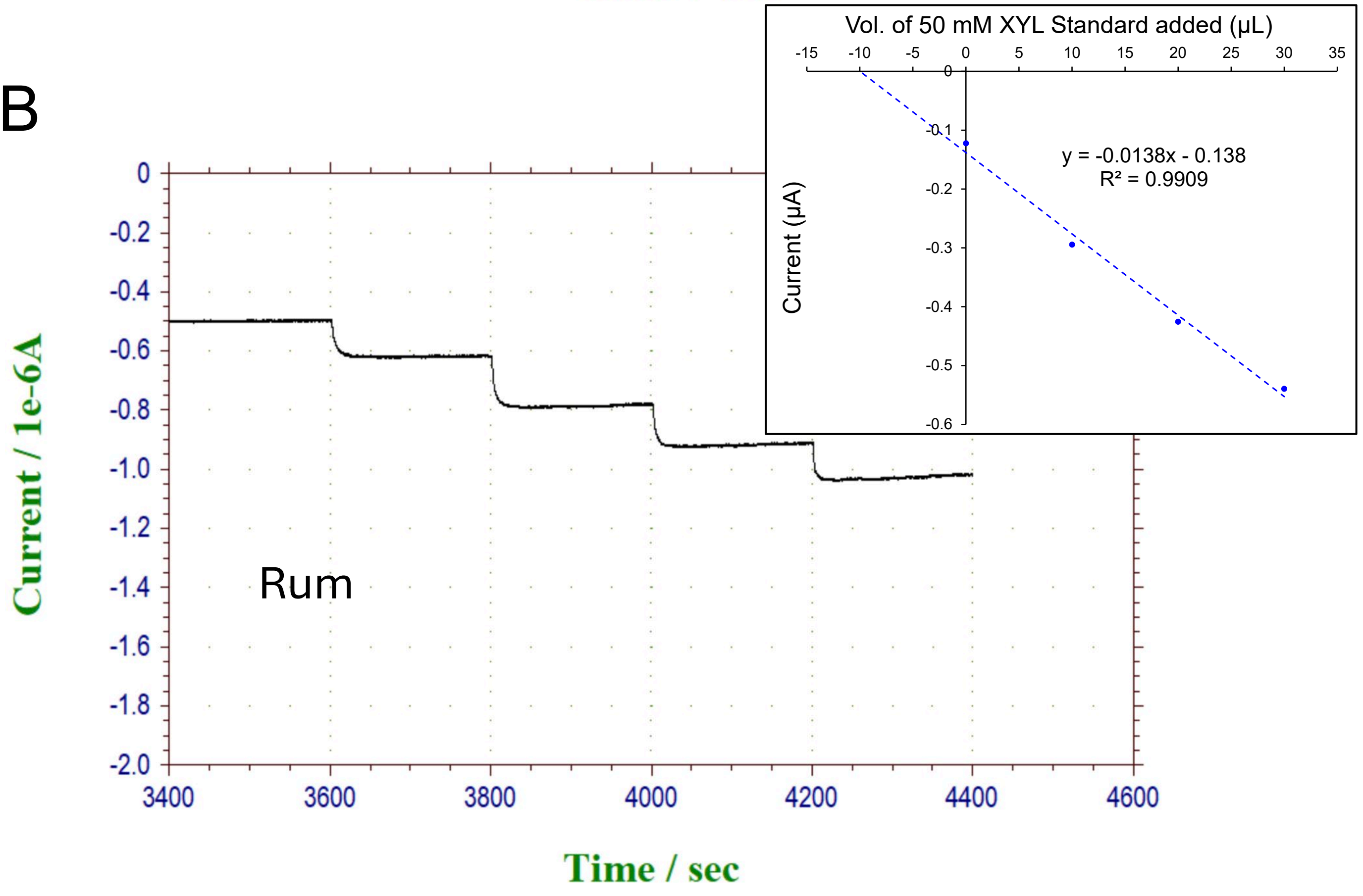

Figure S17. Representative amperometric I-t curve and typical standard addition plot (**inset**) for XYL analysis in (**A**) gin and (**B**) rum.

Table S-1. Comparison of XYL Detection Techniques in Literature

Technique	Method	Material or Electrode	As Reported Sensitivity (units vary)	Approx. Reported LOD (units vary)	Application	Ref
Echem	DPV	GCE	0.4 μA/(μg/mL·cm^2)	120 nM (0.03 ppm)	Sexual assault (urine)	1
Echem	DPV	MWCNT (carbon paste)	2.6 [a] μA/(ng/mL)	4.8 nM (0.001 ppm)	Blood Serum	2
Echem	Volt	Graphene (nanoplates)	12.5 μA/(μg/mL·cm^2)	0.1 mg/L (0.1 ppm)	Sexual assault (beverages)	3
Echem	DPV	Graphene (nanocoral)	10.4 μA/(μg/mL·cm^2)	0.03 mg/L (0.03 ppm)	Sexual assault (beverages)	4
Optical	UV-Vis	Polymers/ Dyes	0.09 a.u./mM	1.36 mM (300 ppm)	Sexual assault (beverages)	16
Echem	SWV	Graphene (laser-scribed)	0.07 [a] μA/μM	140 nM (0.03 ppm)	Sexual assault (beverages)	22
Echem	Amp	GCE (modified)	0.06 μA/(μM·cm^2)	1-10 ppm	Sexual assault (beverages)	This work

Notes: Echem = electrochemistry; DPV = differential pulse voltammetry; Volt = stripping voltammetry; SWV = square wave voltammetry Optical = spectroscopy; UV-Vis = ultraviolate-visible spectroscopy; Amp = amperometry; LOD = limit of detection; [a] Sensitivity not normalized to surface area (geometric or real).

References from Table:

1. Mendes, L.F.; Silva, A.R.S.E.; Bacil, R.P.; Serrano, S.H.P.; Angnes, L.; Paixao, T.R.L.C.; de Araujo, W.R. Forensic electrochemistry: Electrochemical study and quantification of xylazine in pharmaceutical and urine samples. *Electrochimica Acta* **2019**, *295*, 726-734, doi:10.1016/j.electacta.2018.10.120.
2. El-Shal, M.A.; Hendawy, H.A.M. Highly Sensitive Voltammetric Sensor Using Carbon Nanotube and an Ionic Liquid Composite Electrode for Xylazine Hydrochloride. *Analytical Sciences* **2019**, *35*, 189-194, doi:10.2116/analsci.18P368.
3. Saisahas, K.; Soleh, A.; Promsuwan, K.; Saichanapan, J.; Phonchai, A.; Sadiq, N.S.M.; Teoh, W.K.; Chang, K.H.; Abdullah, A.F.L.; Limbut, W. Nanocoral-like Polyaniline-Modified Graphene-Based Electrochemical Paper-Based Analytical Device for a Portable Electrochemical Sensor for Xylazine Detection. *Acs Omega* **2022**, *7*, 13913-13924, doi:10.1021/acsomega.2c00295.
4. Saisahas, K.; Soleh, A.; Promsuwan, K.; Phonchai, A.; Sadiq, N.S.M.; Teoh, W.K.; Chang, K.H.; Abdullah, A.F.L.; Limbut, W. A portable electrochemical sensor for detection of the veterinary drug xylazine in beverage samples. *J Pharmaceut Biomed* **2021**, *198*, doi:10.1016/j.jpba.2021.113958.
16. Marroquin-Garcia, R.; van Wissen, G.; Cleij, T.J.; Eersels, K.; van Grinsven, B.; Diliën, H. Single-use dye displacement colorimetry assay based on molecularly imprinted polymers: Towards fast and on-site detection of xylazine in alcoholic beverages. *Food Control* **2024**, *161*, doi:10.1016/j.foodcont.2024.110403.
21. de Lima, L.F.; de Araujo, W.R. A highly efficient and portable laser-scribed graphene-based electrochemical system for forensic-oriented determination of acepromazine. *Anal Methods-Uk* **2023**, *15*, 4467-4476, doi:10.1039/d3ay00815k.
